# Immunogenicity and tolerability of a heterologous booster typhoid conjugate vaccine (TCV) five to six years after primary vaccination in Burkinabe Children: An open-label, non-randomized follow-up of a phase 2 trial cohort

**DOI:** 10.1371/journal.pgph.0006855

**Published:** 2026-11-13

**Authors:** Jean W. Sawadogo, Alimatou Hema, Diarra Amidou, Jean Moise Kaboré, Denise Hien, Ludovic Kouraogo, Aminata R. Zou, Amidou Z. Ouédraogo, Alfred B. Tiono, Shrimati Datta, Marcela F. Pasetti, Kathleen M. Neuzil, Sodiomon B. Sirima, Alphonse Ouédraogo, Matthew B. Laurens

**Affiliations:** 1 Groupe de Recherche Action en Santé (GRAS), Ouagadougou, Burkina Faso; 2 Center for Vaccine Development and Global Health (CVD), University of Maryland School of Medicine, Baltimore, Maryland, United States of America; University of Alabama at Birmingham, UNITED STATES OF AMERICA

## Abstract

Typhoid fever remains a major public health challenge in low- and middle-income countries. In 2018, the World Health Organization recommended a single dose of typhoid conjugate vaccine (TCV) for routine immunization in endemic settings; however, evidence to inform booster dosing remains limited. This study evaluated the immunogenicity and safety of a heterologous booster strategy using a Vi capsular polysaccharide-CRM197 TCV (Vi-CRM) administered 5–6 years after primary vaccination with a Vi capsular polysaccharide tetanus toxoid TCV (Vi-TT). In this open-label, non-randomized follow-up study, children previously enrolled in a Phase 2 randomized trial in Ouagadougou, Burkina Faso, were re-enrolled. Participants who had received TCV at 9–11 or 15–23 months of age received a Vi-CRM booster at 6–7 years of age (Booster-TCV group), while control participants received their first TCV dose at the same age (1st-TCV group). Group assignment reflected allocation in the parent trial; no new randomization was performed. Serum anti-Vi IgG concentrations were measured before vaccination and 28 days afterward. Seroconversion was defined as a ≥ 4-fold increase from baseline. Solicited and unsolicited adverse events (AEs) and serious adverse events (SAEs) were recorded. Among 147 enrolled children, 87 received a booster dose and 60 received a first TCV dose. Baseline anti-Vi IgG geometric mean titers (GMTs) were higher in the Booster-TCV group than in the 1st-TCV group (21.5 [95% CI, 17.2-26.8] vs 5.5 [95% CI, 4.5-6.8] EU/mL). By day 28, GMTs increased to 5140.0 (95% CI, 4302.0-6141.3) and 2084.8 (95% CI, 1724.4-2520.5) EU/mL, respectively, and all participants with evaluable samples seroconverted. Solicited reactions were infrequent and mild, and no SAEs occurred through day 28. These findings demonstrate robust immunogenicity and short-term tolerability of heterologous TCV boosting; however, because no correlate of protection exists for TCVs, the results do not establish sustained or post-booster clinical protection.

**Trial registration:** ClinicalTrials.gov NCT07624175 **(registered 2026-06-03); parent trial NCT03614533**

## Introduction

Typhoid fever remains a major global public health challenge, particularly in low- and middle-income countries where access to safe water, sanitation, and healthcare is limited. Despite a marked decline in disease burden over the past three decades, estimates indicate that typhoid fever causes more than 7 million cases and approximately 93,000 deaths annually worldwide [1–3]. Children, especially those under five years of age and those of school age, are disproportionately affected [4], underscoring the urgent need for effective preventive measures, including vaccination alongside improvements in water and sanitation.

Typhoid fever, caused by *Salmonella enterica* serovar Typhi, can lead to serious complications including intestinal perforation, gastrointestinal hemorrhage, and neurological complications, particularly when effective treatment is delayed [5]. The ever-growing problem of antimicrobial resistance further complicates treatment [6] and can result in treatment failure, prolonged illness, greater healthcare costs, and potentially worse clinical outcomes.

Typhoid conjugate vaccine (TCV) provides protection against *Salmonella* Typhi and is recommended by the World Health Organization (WHO) for routine immunization in endemic areas. Compared with older polysaccharide vaccines [7], TCV offers important advantages including more durable antibody responses and safe administration to infants as young as six months of age [8–10]. Among TCV products, the Vi-tetanus toxoid conjugate vaccine (Vi-TT) and diphtheria toxin variant conjugate vaccine TYPHIBEV Vi-CRM (Biological E., Ltd., Hyderabad, India), have demonstrated strong immunogenicity and safety in Africa and Asia [11–13]. Nevertheless, data remain limited regarding optimal dosing schedules and the duration of protective immunity.

Multiple clinical trials have demonstrated TCV safety, immunogenicity, and efficacy, supporting vaccine introductions in multiple countries. Ultimately, these data, together with support from Gavi, the Vaccine Alliance, led to TCV being added to routine Expanded Programme on Immunisation (EPI) vaccination schedules for pediatric populations in endemic countries [8,9]. Understanding both the duration of protection conferred by TCV and the need for and timing of booster doses is central to typhoid prevention and control.

In Burkina Faso, an RCT demonstrated safety and immunogenicity of the Vi-TT TCV product administered with Expanded Program on Immunization (EPI) vaccines in children aged 9–11 and 15–23 months of age [8,9]. This study cohort provided a unique opportunity to assess the safety and immunogenicity of a booster TCV dose with a different TCV product (Vi-CRM), administered approximately 5–6 years later. Evidence on antibody persistence and responses to booster vaccination may inform future evaluations of TCV dosing strategies in endemic settings. The objectives of the present study were to describe the persistence of anti-Vi IgG 5–6 years after Vi-TT vaccination in early childhood, to compare the anti-Vi IgG response to Vi-CRM between previously Vi-TT-vaccinated and TCV-naïve children, and to describe the safety and reactogenicity of Vi-CRM in both groups.

## Materials and methods

### Ethics statement

The study protocol (version 1.0, 9 March 2024) was approved by Comité d’éthique pour la recherche en santé (CERS) of Burkina Faso (approval no. 2024-04-95 on 03 April 2024). All participants were younger than 18 years. Written informed consent was obtained from a parent or legal guardian of each participant before any study procedure. Child assent was not required because participants were aged 6–7 years, below the age at which the approving ethics committee required assent. Parents or guardians received 7,500 FCFA (approximately US$15) per clinic visit as compensation for transport and time, as specified in the approved protocol. The study was conducted in accordance with the Declaration of Helsinki and Good Clinical Practice.

### Study design

This open-label, non-randomized, prospective interventional follow-up study was conducted at a school-aged child outpatient clinic at Schiphra Protestant Hospital, an urban hospital in Ouagadougou, Burkina Faso. It followed a Phase 2 randomized, double-blind, controlled trial evaluating the safety and immunogenicity of Vi-TT among children younger than 2 years of age in Ouagadougou, and a subsequent assessment of the durability of vaccine-induced anti-Vi IgG and IgA responses in children immunized with TCV at the routine 15-month visit in Burkina Faso [8–10].

### Trial registration

The parent trial was registered at ClinicalTrials.gov under identifier NCT03614533 (https://clinicaltrials.gov/study/NCT03614533). This follow-up booster study enrolled participants from that registered trial and was conducted as a prospective interventional study with institutional ethics approval, registered at ClinicalTrials.gov under identifier NCT07624175 (https://clinicaltrials.gov/study/NCT07624175).

### Participants

Details of the original studies are published [8,9]. In brief, we recruited, enrolled, and vaccinated two cohorts of healthy children in 2018–2019: 100 aged 9–11 months and 150 aged 15–23 months. The younger cohort was randomized 1:1 to receive either Vi-TT (Group 1) or an inactivated polio vaccine (IPV; Group 2) as the control. In the older cohort, participants were randomized into one of three groups: Vi-TT and IPV with delayed meningococcal A conjugate vaccine (MCV-A, Group 1), Vi-TT and MCV-A (Group 2), or MCV-A and IPV (Group 3). All vaccines were administered intramuscularly along with routine measles-rubella (MR) and yellow fever (YF) vaccines.

This study re-enrolled children who had previously participated in the phase 2 trial in Ouagadougou. In the current study, participants who originally received Vi-TT were administered a second dose of TCV using the Vi-CRM product, while those who had not previously received TCV were given their first dose at 6–7 years of age using the Vi-CRM product.

Families of children who had previously participated in the TCV study were contacted by phone call or via community-based health workers serving as guides. Detailed study information was provided to these families through individual or group discussions. Parents or guardians received both oral and written explanations, in local and French languages, of study procedures and provided written informed consent in a private setting before enrollment and blood sample collection.

Eligibility criteria specified in protocol included prior participation in the 2018–2019 Phase 2 trial and parent or legal guardian consent. Some potentially eligible participants had relocated outside the study area and were not re-enrolled because transportation from outside the study area was not provided. Residence within the study area was not specified as an eligibility criterion in protocol version 1.0. Participants were excluded if they received blood products within the previous six months, received TCV outside the study (e.g., through a vaccination campaign or routine immunization), or had any condition deemed by the investigator to potentially interfere with vaccine evaluation or pose a health risk. Additionally, temporary exclusion criteria were applied to participants who met any of the following conditions, with reassessment after 48 hours: reported fever within 24 hours before vaccination and/or use of any antipyretic medications within 4 hours before vaccination.

### Group assignment and masking

No randomization was performed in the present study. Participants were assigned to the Booster-TCV or 1st-TCV group according to their allocation in the parent randomized trial (Vi-TT or control vaccine). The comparison is therefore based on historical randomized allocation among children who could be recontacted and re-enrolled. The current Vi-CRM vaccination was administered open-label; however, parent trial vaccination assignment remained masked to study personnel other than the study biostatistician.

### Procedures

TYPHIBEV Vi-CRM (Biological E., Ltd., Hyderabad, India), contains 25 μg of Vi polysaccharide conjugated to a protein carrier, which is a variant of diphtheria toxin (CRM197). Vi-CRM was administered intramuscularly in the left upper deltoid. Vaccinations were performed at the outpatient clinic of Schiphra Protestant Hospital in Ouagadougou, Burkina Faso. Trained local study personnel not involved in participant follow-up or adverse event (AE) monitoring administered the injections. On study Day 0, each participant underwent blood sampling and subsequently received the Vi-CRM vaccine. After vaccination, participants were monitored onsite for serious reactions, and via phone on Day 7 post-vaccination to inquire about local and systemic AEs that occurred each day since vaccination. Study personnel were available for unscheduled visits during the 28-day follow-up period. Unsolicited AEs and serious AEs (SAEs) were recorded until Day 28. Adverse events were graded according to the United States Food and Drug Administration (FDA) guidelines for vaccine clinical trials (US Food and Drug Administration, 2007). Blood samples were collected for immunogenicity assessment on Days 0 and 28. Concomitant medications received from day 0 to day 28 were recorded but were not analyzed as a separate study outcome. Anti-Vi IgG responses were measured using the VaccZyme Salmonella Typhi Vi Immunoglobulin G (IgG) ELISA kit (The Binding Site Group Ltd, Birmingham, UK), reported in EU/mL, with an assay detection limit of 7.4 EU/mL. All anti-Vi IgG measurements used in the longitudinal comparisons were performed using the same assay at the Applied Immunology Laboratory, Center for Vaccine Development and Global Health, University of Maryland School of Medicine, Baltimore, Maryland, USA. Although the protocol specified measurement of both anti-Vi IgG and IgA, the investigative team subsequently decided, after consultation with experts in the field, to limit the immunogenicity assessment to anti-Vi IgG. Anti-Vi IgA was therefore not measured. Data analysis was performed at the University of Maryland, Baltimore in Baltimore, Maryland, United States of America.

### Outcomes

The primary estimand was the ratio of day 28 anti-Vi IgG GMTs after Vi-CRM vaccination between children previously vaccinated with Vi-TT at 9–11 or 15–23 months of age (Booster-TCV group) and children who had not previously received TCV (1^st^-TCV group). The primary outcome measure was the serum anti-Vi IgG concentration 28 days after Vi-CRM vaccination, summarized as the geometric mean titer (GMT) in each group; the primary comparison was between the Booster-TCV and 1st-TCV groups. Secondary immunogenicity measures were the pre-vaccination (day 0) GMT, the geometric mean fold rise (GMFR) from day 0 to day 28, and seroconversion, defined as a ≥ 4-fold rise in anti-Vi IgG from the day-0 (pre-Vi-CRM) sample to day 28. For descriptive purposes, the fold rise and seroconversion were also calculated from the pre-vaccination sample of the parent trial (infant baseline) to day 28 after Vi-CRM; these cumulative measures are labelled as such.

Secondary outcomes included TCV safety and reactogenicity, assessed via solicited and unsolicited AEs. Solicited local reactions (pain/tenderness, swelling, erythema) and solicited systemic reactions (fever, feverishness, myalgia, arthralgia, malaise) occurring from day 0 to day 7 were collected systematically by structured telephone interview on day 7. Unsolicited AEs and SAEs from day 0 to day 28 were collected non-systematically, through spontaneous reporting including at unscheduled visits, and recorded up to the day-28 visit. The relationship of each AE to vaccination was assessed by the investigator.

### Patient and public involvement

Community-based health workers assisted in recontacting participating families. As specified in the protocol, summary results will be shared with participating communities through community meetings after completion of the clinical study report.

### Statistical analysis

All data were analyzed with SAS software Version 9.4 (2013 SAS Institute Inc., Cary, NC, USA).

Anti-Vi IgG values below the assay limit of detection (7.4 EU/mL) were assigned half that value (3.7 EU/mL). Antibody concentrations were log-transformed; GMTs, GMFRs and their 95% confidence intervals (CIs) were calculated on the natural-log scale and back-transformed, and figures display log10-transformed values. Within-group changes were compared with paired t-tests and between-group differences with two-sample t-tests with unequal variances on log-transformed values. Proportions (seroconversion and adverse events) are presented with exact (Clopper-Pearson) 95% CIs. Between-group comparisons are unadjusted; no adjustment was made for age at primary vaccination or for multiple comparisons, and p-values are descriptive. No interim analyses were planned or performed. Although the protocol specified that a separate Statistical Analysis Plan would be finalized before analysis, analyses were conducted according to the statistical methods described in the protocol, and no standalone SAP was developed.

### Sample size determination

Of 250 participants in the parent trial who were potentially eligible for follow-up, 148 had received Vi-TT, and 102 had received control vaccine. Assuming that 25% could not be contacted or recruited, the protocol anticipated follow-up of approximately 187 participants. No formal power calculation was performed for the present study.

### Analysis sets

Descriptive statistics were used to summarize baseline demographics, with age presented as mean ± standard deviation and sex as proportions. The intention-to-treat (ITT) population, as defined in the protocol, comprised all 147 enrolled participants, all of whom were vaccinated. Safety analyses used endpoint-specific denominators: participants contacted on day 7 for solicited reactions and all vaccinated participants for unsolicited AEs and SAEs through day 28. The incidence of local and systemic AEs within seven days post-vaccination was compared between the Booster-TCV and 1st-TCV groups. The incidence of unsolicited AEs and SAEs within 28 days of vaccination were also compared between the same groups.

Immunogenicity was analyzed within the ITT population using all available samples at each timepoint (available-case analysis): the day-28 immunogenicity population comprised participants with a day-28 sample, and the durability sub-study population comprised participants of the parent trial’s durable-antibody sub-study with a 30–35-month sample. Antibody distributions were visualized and reported using sample size, geometric mean titers, 95% confidence intervals, and p-values.

A multi-timepoint descriptive immunogenicity analysis was conducted within the ITT population using all available samples at each timepoint. The 30–35-month measurements were available only for participants in the durability sub-study; therefore, participants contributing observations at different timepoints overlapped but were not identical. A previous investigation assessed anti-Vi IgG and IgA antibodies 30–35 months after vaccination in a subset of participants from a clinical trial in which TCV had been co-administered with the routine vaccine at the 15-month vaccination visit [10]. In the present analysis, antibody titers were compared from baseline to 30–35 months after primary TCV administration, and from baseline to 28 days post-booster dosing at 6–7 years.

## Results

### Baseline characteristics

Of 250 children originally vaccinated between 9 and 23 months of age in the randomized controlled trial, 157 (62.8%) were successfully recontacted in 2024, and 152 came to the study site for potential enrollment. Following five refusals, 147 participants were enrolled and vaccinated between August and October 2024: 87 in the Booster-TCV group and 60 in the 1st-TCV group. The proportion re-enrolled was identical according to parent-trial treatment assignment: 87/148 (58.8%) previous Vi-TT recipients and 60/102 (58.8%) previous control recipients were enrolled. Re-enrollment was more frequent among children originally enrolled at age 15–23 months than among those enrolled at age 9–11 months (99/150 [66.0%] vs 48/100 [48.0%], p = 0.005), but did not differ materially by sex, height, weight, or temperature at parent-trial enrollment (S2 Table). Some potentially eligible children who had relocated outside the study area were not re-enrolled because transportation from their new locations was not provided. Residence within the study area was not specified as an eligibility criterion in protocol version 1.0. The two groups showed comparable demographic profiles, with a mean age of 6.8 years (SD 0.3) overall; 48/87 (55.2%) participants in the Booster-TCV group and 27/60 (45.0%) in the 1st-TCV group were male (Table 1). Among 147 participants, 88 (59.9%) had participated in the durable anti-Vi IgG sub-study, including 52 in the Booster-TCV group and 36 in the 1st-TCV group (Fig 1 and Table 1).

**Table 1:** Baseline socio-demographic characteristics.

	Booster TCV n = 87	First TCV n = 60	Overall n = 147
Age at enrollment			
Mean (±SD)	6.8 (0.3)	6.7 (0.3)	6.8 (0.3)
Sex			
Female n (%)	39 (44.8)	33 (55.0)	72 (49.0)
Male n (%)	48 (55.2)	27 (45.0)	75 (51.0)

**Fig 1:**
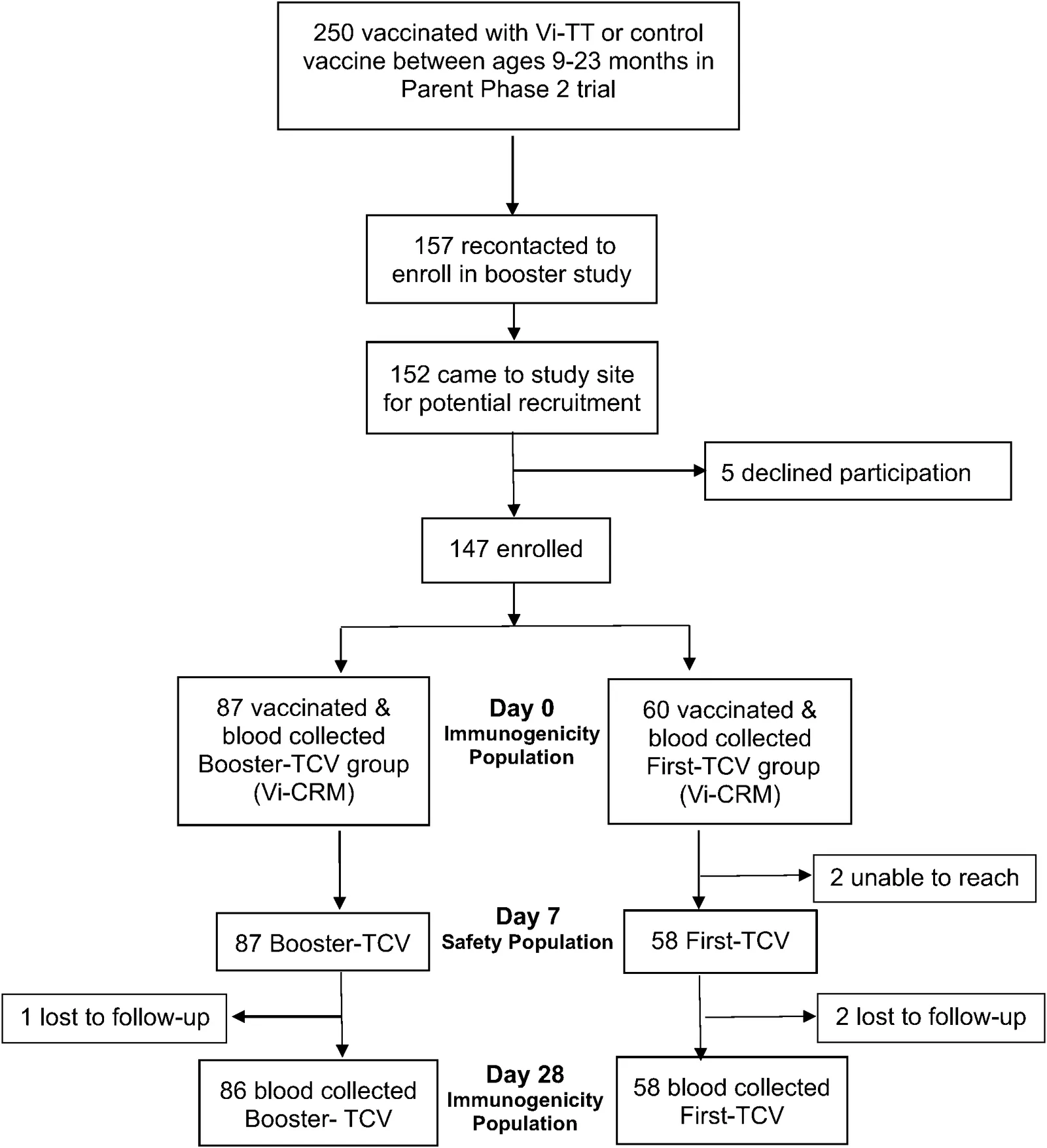
Participant flow through enrollment and follow‑up in the typhoid conjugate vaccine booster study.

### Reactogenicity and short-term safety

Solicited reactions were assessed in 87 Booster-TCV and 58 1st-TCV participants (two 1st-TCV participants could not be reached on day 7), and unsolicited AEs and SAEs in all 87 and 60 vaccinated participants (Fig 1). Local injection site reactions within seven days post-vaccination were reported exclusively in the Booster-TCV group, affecting 4/87 participants (4.6%; 95% CI: 1.3-11.4; Table 2). These reactions were mild pain or tenderness at the injection site. No local reactions were observed in the 1st-TCV group (0/58).

**Table 2:** Summary of reactogenicity and safety parameters (adverse events) by vaccine group in the safety population after Vi-CRM vaccination.

		Booster-TCV n = 87	1st-TCV n=58†/ 60‡
Local Reactions at injection site (Day 0–7)			
	**Pain/tenderness**	4 (4.6, 1.3-11.4) Mild (4) on Day 1	0 (0.0, 0.0-6.2)
	**Swelling**	0 (0.0, 0.0-4.2)	0 (0.0, 0.0-6.2)
	**Erythema**	0 (0.0, 0.0-4.2)	0 (0.0, 0.0-6.2)
	**Any local reaction**	4 (4.6, 1.3-11.4)	0 (0.0, 0.0-6.2)
Systemic Reactions (Day 0–7)			
	**Fever**	2 (2.3, 0.3-8.1) Day 2 (1), Day 6 (1)	4 (6.9, 1.9-16.7) Day 2 (1), Day 3 (3)
	**Feverishness**	0 (0.0, 0.0-4.2)	0 (0.0, 0.0-6.2)
	**Myalgia**	0 (0.0, 0.0-4.2)	0 (0.0, 0.0-6.2)
	**Arthralgia**	0 (0.0, 0.0-4.2)	0 (0.0, 0.0-6.2)
	**Malaise**	0 (0.0, 0.0-4.2)	0 (0.0, 0.0-6.2)
	**Any systemic reaction**	2 (2.3, 0.3-8.1)	4 (6.9, 1.9-16.7)
Unsolicited adverse events (Day 0–28)			
	**Related**	0 (0.0, 0.0-4.2)	1 (1.7, 0.04-8.9)
	**Not related**	23 (26.4, 17.6-37.0)	18 (30.0, 18.9-43.2)
	**Any unsolicited adverse event**	23 (26.4, 17.6-37.0)	19 (31.7, 20.3-45.0)

Data are n (%, 95% CI). n = number of participants. CI = confidence interval. Vi-CRM: Vi polysaccharide diphtheria toxin conjugated vaccine. †Solicited reactions (day 0–7): two 1st-TCV participants could not be reached on day 7. ‡Unsolicited adverse events (day 0–28): all 60 vaccinated 1st-TCV participants. 95% CIs are exact (Clopper-Pearson).

Systemic AEs were slightly more frequent in the 1st-TCV group, with an incidence of 4/58 (6.9%; 95% CI: 1.9-16.7), compared to 2/87 (2.3%; 95% CI: 0.3-8.1) in the Booster-TCV group (Table 2), with widely overlapping confidence intervals; the study was not powered to compare reactogenicity between groups. Fever was the only systemic reaction reported (two Booster-TCV and four 1st-TCV participants). No other systemic symptoms were noted in either group.

Unsolicited adverse events (AEs) occurring after vaccination were reported at comparable rates in both study groups: 23/87 (26.4%; 95% CI: 17.6-37.0) in the Booster-TCV group and 19/60 (31.7%; 95% CI: 20.3-45.0) in the 1st-TCV group. Most events were mild or moderate. One unsolicited AE in the 1st-TCV group was assessed as related to vaccination and none in the Booster-TCV group (Table 2). Rhinitis and uncomplicated malaria were the most frequently reported conditions (S1 Table). No participant was excluded from study participation because of concomitant medication use, and no concomitant vaccinations occurred during the 28-day follow-up period. No serious adverse event (SAE) was recorded through day 28. All unsolicited AEs resolved without sequelae.

### Immunogenicity

#### IgG geometric mean titer and seroconversion at Day 28 after vaccination.

At baseline just before Vi-CRM vaccination, children in the Booster-TCV group exhibited significantly higher anti-Vi IgG geometric mean titers (GMT) compared to those in the 1st-TCV group (21.5 EU/mL; 95% CI: 17.2-26.8 vs. 5.5 EU/mL; 95% CI: 4.5-6.8), consistent with persistent vaccine-associated antibody, although natural exposure or cross-reactive antigenic exposures may also have contributed. By day 28, GMTs increased to 5140.0 EU/mL (95% CI: 4302.0-6141.3) in the Booster-TCV group and 2084.8 EU/mL (95% CI: 1724.4-2520.5) in the 1^st^-TCV group, corresponding to an unadjusted GMT ratio of 2.5 (95% CI 1.9-3.2-) (n = 86 and n = 58, respectively; Fig 2)

**Fig 2:**
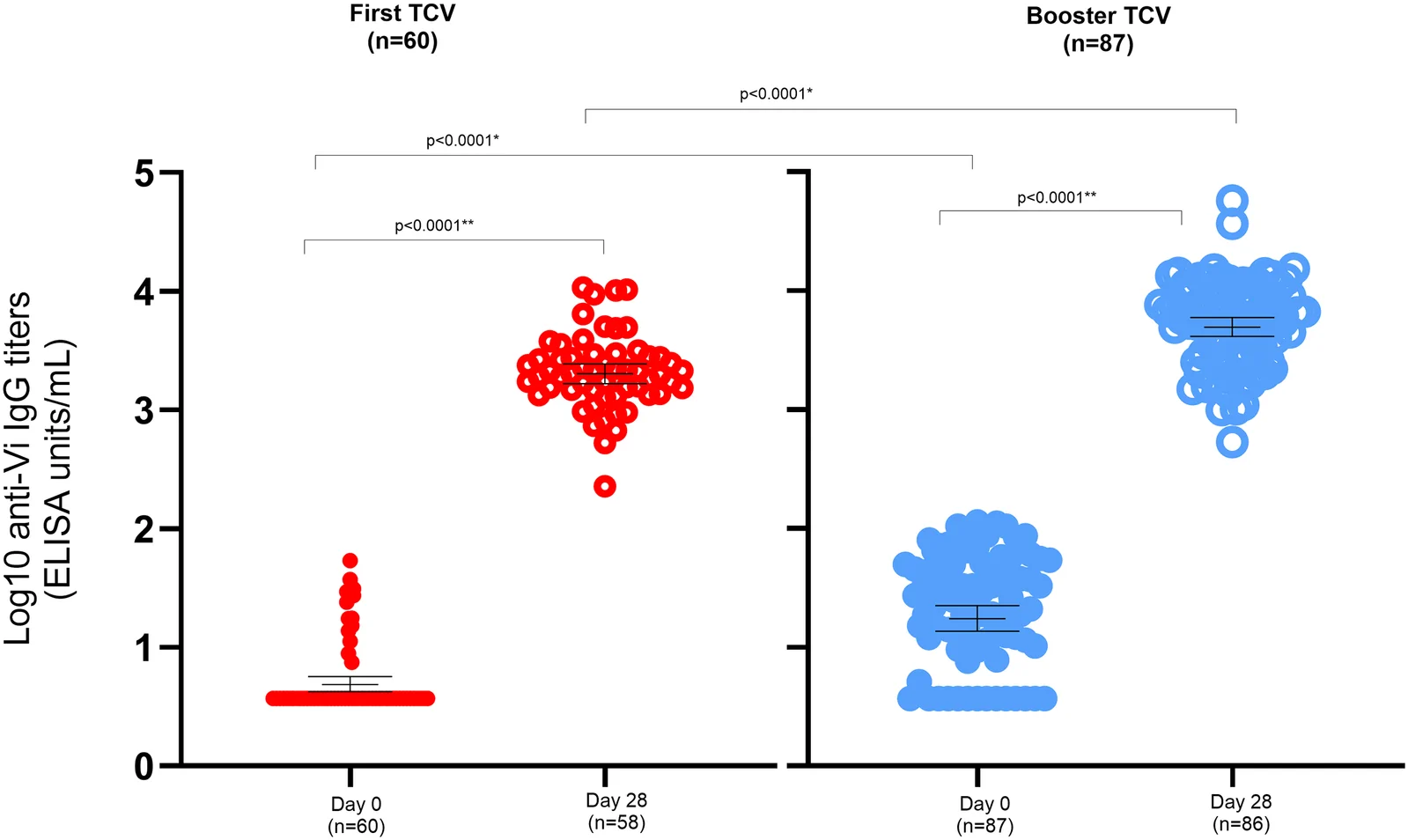
Anti-Vi IgG before (Day 0) and 28 days after Vi-CRM vaccination in the intention to treat protocol population. Circles represent the log10 transformed antibody titer result for each individual participant in each corresponding vaccine group. Bar in the middle of the individual participant results for each time point represents the geometric mean antibody titer value on log10 scale with 95% confidence interval. * Using two sample t-test with unequal variances on log10 transformed data. ** Using paired t-test on log10 transformed data. Between-group comparison at Day 28 is unadjusted.

Seroconversion from the pre-Vi-CRM baseline (≥4-fold rise from day 0 to day 28) occurred in all participants with day-28 samples: 86/86 (100.0%; 95% CI 95.8-100.0) in the Booster-TCV group and 58/58 (100.0%; 95% CI 93.8-100.0) in the 1st-TCV group. Measured from the infant baseline of the parent trial, the cumulative rise was 805.6-fold (95% CI 561.4-1158.9) in the Booster-TCV group and 331.5-fold (236.3-465.0) in the 1st-TCV group, and cumulative seroconversion was 86/86 (100.0%) and 57/58 (98.3%), respectively (Table 3).

**Table 3:** Anti-Vi IgG before and after first IPV or Vi-TT vaccination and before and after Vi-CRM vaccination (available-case analysis within the intention-to-treat population).

	Booster-TCV		1st-TCV	
	*n or n/N*	*Mean or % (95% CI)*	*n* *n/N*	*Mean or % (95% CI)*
**GMT (EU/mL)**				
**Initial Vaccination:**		Vi-TT		IPV
**Day 0**	87	6.3 (4.9-8.2)	60	6.3 (4.6-8.7)
**Day 28**	86	2213.1 (1533.3-3194.4)	60	6.1 (4.7-7.8)
**Months 30–35**	52	81.0 (61.7-106.4)	36	6.0 (4.4-8.1)
**Current Vi-CRM Vaccination**^**§**^:		Vi-CRM		Vi-CRM
**Day 0**	87	21.5 (17.2-26.8)	60	5.5 (4.5-6.8)
**Day 28**	86	5140.0 (4302.0-6141.3)	58	2084.8 (1724.4-2520.5)
**GMFR**				
**Pre-Vi-CRM Day 0 to Vi-CRM Day 28**	86	238.5 (184.0-309.2)	58	375.5 (305.1-462.1)
**Cumulative parent-trial Day 0 to Vi-CRM Day 28**	86	805.6 (561.4-1158.9)	58	331.5 (236.3-465.0)
**Seroconversion ≥ 4-fold increase from**				
**Pre-Vi-CRM Day 0 to Vi-CRM Day 28**	86/86	100.0 (95.8-100.0)	58/58	100.0 (93.8-100.0)
**Cumulative parent-trial Day 0 to Vi-CRM Day 28**	86/86	100.0 (95.8-100.0)	57/58	98.3 (90.8-100.0)

n = number of participants. CI = confidence interval. Vi-CRM: Vi polysaccharide diphtheria toxin conjugated vaccine. Vi-TT: Vi polysaccharide tetanus toxoid conjugate vaccine. IPV: inactivated polio virus vaccine. GMT: geometric mean titers. EU: ELISA units. GMFR: geometric mean fold rise. ^§^Current Vi-CRM vaccination received 2.5 + years post Months 30–35 timepoint.

Results at each timepoint are based on available samples. Participants contributing observations at different timepoints overlapped but were not identical.

In exploratory analyses stratified by age at primary vaccination (S3 Table), pre-Vi-CRM GMTs in the Booster-TCV group were 12.0 EU/mL (95% CI 8.0-18.2) among children vaccinated at 9–11 months (n = 27) and 27.8 EU/mL (21.8-35.5) among those vaccinated at 15–23 months (n = 60), compared with 4.8 EU/mL (3.5-6.4) and 5.9 EU/mL (4.5-7.7) in the corresponding 1st-TCV strata. Day-28 GMTs after Vi-CRM were 3573.4 EU/mL (2716.2-4701.1) and 6070.4 EU/mL (4877.6-7554.9) in the two Booster-TCV strata and 1844.9 EU/mL (1220.0-2790.0) and 2223.3 EU/mL ([1811.1-2729.4]) in the 1st-TCV strata. Seroconversion from the pre-Vi-CRM baseline was 100% in all four strata.

#### Longitudinal anti-Vi IgG responses across available study timepoints.

As shown in Fig 3 and Table 3, a multi-timepoint analysis of anti-Vi IgG responses was conducted among participants in the durable antibody sub-study of the randomized controlled trial. Available-case analyses describe antibody concentrations after initial Vi-TT vaccination at 9 or 15 months of age through post-booster administration at 6–7 years. At baseline (before the initial Vi-TT vaccination), anti-Vi IgG GMTs were comparable between the Booster-TCV and 1st-TCV groups. After primary vaccination, GMTs in the Booster-TCV group rose significantly to 2213.1 EU/mL (95% CI: 1533.3-3194.4) by day 28. A gradual decline was observed over time, with GMTs decreasing to 81.0 EU/mL (95% CI: 61.7-106.4) at the 30–35-month timepoint. At enrollment into the booster study, GMTs in the Booster-TCV group were 21.5 EU/mL (95% CI: 17.2-26.8), consistent with persistent anti-Vi IgG concentrations at the group level. Post-booster, anti-Vi IgG GMTs increased markedly to 5140.0 EU/mL (95% CI: 4302.0-6141.3) by day 28, consistent with a strong anamnestic response. In the 1st-TCV group, anti-Vi IgG GMTs remained low from infancy until the first TCV dose at age 6–7 years, after which titers rose to 2084.8 EU/mL (95% CI: 1724.4-2520.5) by day 28.

**Fig 3:**
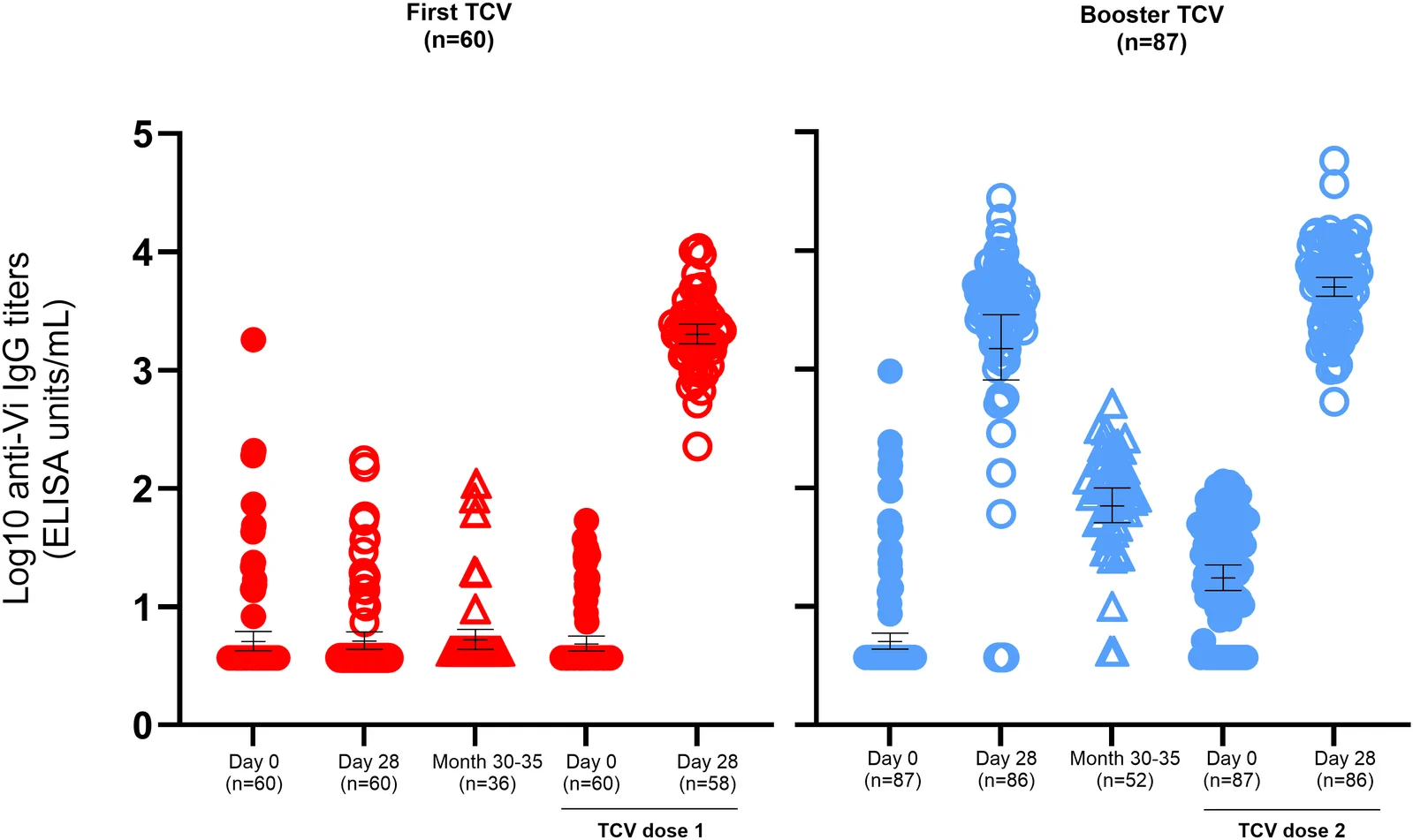
Anti-Vi IgG before (Day 0) and after (Day 28 and 30-35 months) first IPV or Vi-TT vaccination and before and after Vi-CRM vaccination in the intention to treat population. TCV dose 1 and dose 2 received 2.5 + years after Month 30–35 timepoint. Circles and triangles represent the log10 transformed antibody titer result for each individual participant in each corresponding vaccine group. Bar in the middle of the individual participant results for each time point represents the geometric mean antibody titer value on log10 scale with 95% confidence interval. Results at each visit are based on all available samples (available-case analysis).

## Discussion

In this study, we assessed the persistence of anti‑Vi IgG responses 5–6 years after primary TCV administration in early childhood, and evaluated the immunogenicity and short-term tolerability of a heterologous booster dose delivered at school age. Anti-Vi IgG remained higher in previously vaccinated children than in TCV-naïve peers 5–6 years after primary vaccination, and a heterologous booster dose elicited a strong anamnestic antibody response, with seroconversion in all participants and no serious adverse events during 28 days of follow-up. These results provide longitudinal evidence on the persistence of anti-Vi IgG responses in children living in a typhoid-endemic setting and provide data relevant to ongoing evaluations of booster vaccination and the durability of vaccine-induced immune responses where typhoid burden remains high. Because no serologic correlate of protection has been established for this vaccination, these findings describe immunogenicity rather than clinical protection.

### Immunogenicity findings in context of prior evidence

The immunogenicity results should be interpreted in the broader context of emerging evidence on the durability of long-term TCV responses. While TCVs elicit robust initial protection, emerging data suggest that long-term efficacy may vary by age at first vaccination. A randomized controlled trial in Malawi demonstrated approximately 80% efficacy of a single TCV dose over at least four years [14]. Exploratory age-stratified analyses suggested greater antibody waning and potentially greater reduction in efficacy among children vaccinated before two years of age, although estimates were imprecise [14,15]. These findings have raised interest in whether booster vaccination may be useful for children vaccinated early in life.

Findings from other settings further highlight this heterogeneity. Long-term data from the Philippines indicate that a TCV booster dose sustained anti-Vi immune responses for at least five years, supporting its potential role in durable typhoid prevention [16]. In contrast, data from Bangladesh indicate a more rapid decline in vaccine-induced protection within 3–5 years after single dose TCV administration [17]. Together, these observations underscore the need to further study the longevity of TCV efficacy across different age groups and epidemiologic contexts, and the potential role of booster dosing in endemic areas.

Additional evidence from Malawi demonstrated that a second dose of Vi-TT administered at 5 years of age following primary vaccination at 9–11 months was well tolerated and elicited a significantly greater immune response than a first dose at 5 years [18]. The magnitude of the booster response observed in that study is broadly similar to that observed in the present study conducted in Burkina Faso using a heterologous Vi-CRM booster. Day-28 anti-Vi IgG concentrations, fold rise and seroconversion after the homologous Vi-TT booster in Malawi were of a similar order of magnitude to those observed here after heterologous Vi-CRM boosting [18]. Because the two studies differed in population, laboratory and booster product, and the present study did not include a homologous booster group, this comparison is descriptive: our data show that a heterologous booster is immunogenic, but they cannot establish equivalence or interchangeability of homologous and heterologous approaches.

The durability of antibody responses observed 5–6 years after primary vaccination is consistent with established immunological principles. Immune responses in infants are often less durable than those generated in later childhood, reflecting developmental differences in B-cell maturation, germinal center reactions, and memory formation [19–21]. Previous work has demonstrated that immune responses to polysaccharide antigens, including Vi, vary substantially by age, and that conjugate vaccines only partially overcome these developmental constraints. The observation of sustained anti-Vi IgG responses several years after primary vaccination in children immunized between 9 and 23 months of age adds important longitudinal data to the growing body of evidence on TCV durability. Part of the difference in pre-booster concentrations may also reflect natural exposure to S. Typhi or cross-reactive antigens in this endemic setting, which could not be distinguished from vaccine-induced persistence.

The marked increase in antibody titers after revaccination aligns well with established principles of immunological memory induced by conjugate vaccines. Protein-polysaccharide conjugate vaccines prime memory B cells that can rapidly expand and differentiate upon antigen re-exposure, generating higher antibody concentrations than those achieved by primary vaccination [22]. This phenomenon has been consistently observed with meningococcal, pneumococcal, and typhoid conjugate vaccines [18,23,24], and is consistent with the strong recall responses observed in the present study.

### Reactogenicity and short-term safety in context

The short-term tolerability findings were consistent with previous studies of TCVs across pediatric age groups [8,9,13]. Both solicited and unsolicited adverse events were predominantly mild and transient, and no SAEs were recorded. These findings are consistent with short-term tolerability of Vi-CRM given as a first or booster dose in school-aged children and add to the global safety database for TCVs, which is informed by clinical trials and post-introduction surveillance from multiple countries [25–27]. With 147 participants and 28 days of follow-up, however, this study could not detect uncommon adverse events.

The reactogenicity profile observed in this study, characterized by mostly mild local reactions and minimal systemic symptoms, is typical of protein-conjugate vaccines [18,28,29]. Previous studies have similarly reported acceptable safety profiles for TCVs administered as either primary or booster doses across age groups [30,31]. Importantly, the present study specifically addresses a gap in the literature by describing the short-term tolerability of long-interval booster dosing administered 5–6 years after primary vaccination.

### Implications for vaccination policy

These findings have important implications for typhoid vaccination strategies in endemic settings. In its August 2026 position paper on typhoid vaccines, the WHO recommends that countries with very high typhoid incidence should consider a booster dose around 5 years of age for children who received a primary TCV dose at 9–24 months of age. In high-incidence settings, a booster dose around 5 years of age may be considered if there is evidence of waning protection. In other epidemiological settings where the primary dose of TCV is administered before 24 months of age, a booster dose may also be considered if any evidence of waning protection is observed in vaccinated cohorts, especially in areas with a high case fatality ratio or high prevalence of antimicrobial resistance [32]. WHO recommends that national decisions regarding booster vaccination consider typhoid incidence, case fatality, antimicrobial resistance patterns, cost-effectiveness, and programmatic feasibility, and that a minimum interval of one year between the primary and booster doses be maintained where possible [32]. If a booster dose is introduced, WHO notes that it may be delivered through health facilities, schools, or community outreach, according to existing infrastructure and considerations of affordability and sustainability. WHO also notes that a booster may not be needed in medium- or high-incidence settings where there is limited evidence of waning protection, particularly when primary vaccination occurs at ≥2 years of age [32]. The present study was not designed to determine whether, when or for whom booster doses are needed, nor their effect on clinical protection or their cost-effectiveness; it provides immunogenicity and short-term tolerability evidence following heterologous TCV boosting at school age.

### Heterologous TCV vaccination strategies and Public Health Significance

In this study, we evaluated a heterologous TCV booster strategy in children primed with Vi-TT before age two and boosted with Vi-CRM at school age, and compared immune responses to those elicited by a single dose of Vi-CRM administered at school age. Baseline anti-Vi IgG levels were higher in booster recipients, consistent with persistence of vaccine-induced antibody. Post-vaccination, both groups exhibited strong antibody responses, with higher day-28 titers in the booster group and seroconversion in all participants in both groups. Both groups tolerated vaccination well, and no SAEs were recorded. These findings demonstrate a strong immunogenic response following a heterologous prime-boost approach administered at school age; whether this translates into extended clinical protection requires efficacy or effectiveness data. Although the present study was not designed to establish interchangeability between TCV products, the 2026 WHO position paper considers licensed WHO-recommended TCVs interchangeable for programmatic use and states that any recommended TCV may be used for primary and booster doses [32]. Given the global burden of typhoid, especially among children in South Asia and sub-Saharan Africa, optimizing the timing and delivery of TCV schedules remains a critical public health priority [33].

### Strengths and limitations

Strengths of this study include long-term follow-up of a well-characterized cohort originally enrolled in a randomized trial, availability of infant pre-vaccination and post-primary antibody measurements for a subset of participants, use of the same anti-Vi IgG ELISA assay in the same laboratory across longitudinal study timepoints, and masking of study personnel to prior vaccination status, which limited bias in adverse event ascertainment.

This study has several limitations. First, the sample size was modest and smaller than anticipated (147 enrolled vs 187 expected), which limits precision and the ability to detect uncommon adverse events. Second, participants were not randomized in the present study; group comparisons rely on allocation in the parent trial among the 58.8% of children who could be re-enrolled. Re-enrollment was identical by original allocation and did not differ by sex or anthropometry, but children from the 15–23-month cohort were more likely to be re-enrolled than those from the 9–11-month cohort. In addition, some potentially eligible children who had relocated outside the study area were not re-enrolled because transportation from their new locations was not provided. Consequently, the re-enrolled cohort may differ from children who were not re-enrolled in mobility, health-care access, exposure history, or other unmeasured characteristics, resulting in potential selection bias. Third, higher pre-booster concentrations in previously vaccinated children may partly reflect natural exposure to S. Typhi or cross-reactive antigens. Interval typhoid illness and exposure were not systematically ascertained, so vaccine-associated antibody persistence could not be distinguished from natural boosting. Fourth, immunogenicity was assessed using anti-Vi IgG, and anti-Vi IgA was not measured; no serologic correlate of protection has been established for typhoid conjugate vaccines and no clinical endpoint was assessed, so antibody persistence and booster responses cannot be interpreted as evidence of sustained protection. Fifth, follow-up after Vi-CRM was limited to 28 days, so the durability of the booster response is unknown. Sixth, there was no homologous (Vi-TT) booster group, so equivalence of homologous and heterologous boosting cannot be assessed. Seventh, analyses stratified by age at primary vaccination are based on small numbers, and comparisons were not adjusted for multiplicity. Finally, this was a single-site study in urban Ouagadougou, which may limit generalizability to other settings and age groups.

## Supporting information

S1 Text Study protocol. (PDF)

S1 Table Summary of unsolicited AEs by vaccine arm in the intention to treat population after Vi-CRM vaccination. (PDF)

S2 Table Enrolled vs. non-enrolled children. (PDF)

S3 Table Immunogenicity by age at primary vaccination. (PDF)

S1 Checklist CONSORT Checklist. (DOCX)

S2 Checklist SPIRIT Checklist. (DOCX)
