# Supplementary material for: Immunogenicity and tolerability of a heterologous booster typhoid conjugate vaccine (TCV) five to six years after primary vaccination in Burkinabe Children: An open-label, non-randomized follow-up of a phase 2 trial cohort
Source: PLOS Glob Public Health. 2026 Nov 13;6(11):e0006855. doi: 10.1371/journal.pgph.0006855 (PMC13766224; doi:10.1371/journal.pgph.0006855)
Supplement: Supplementary file 1 [file pgph.0006855.s001.pdf]

Immunogenicity and Tolerability of Typhoid Conjugate Vaccine (TCV) 5-6 Years After Routine Vaccines Administered With or Without TCV among Children Younger than 2 Years in Ouagadougou, Burkina Faso

Sponsor: Center for Vaccine Development and Global Health (CVD), University of Maryland School of Medicine, Baltimore, MD, USA

Funding Agency: Center for Vaccine Development and Global Health, University of Maryland School of Medicine, Baltimore, MD, USA

Medical Investigator Responsible for Participants: Alphonse Ouedraogo, MD, PhD, Groupe de Recherche Action en Santé (GRAS)

Sponsor Investigator: Kathleen Neuzil, MD, MPH

Co-Investigators: Sodiomon B. Sirima, MD, PhD, Alfred B. Tiono MD, PhD, Issa Nébié Ouédraogo MsC, PhD, Matthew Laurens, MD, MPH

Version Number: 1.0

Date: 09 March 2024

Statement of Compliance

This study will be carried out in accordance with the protocol, the International Conference on Harmonization Good Clinical Practice E6 (ICH-GCP); U.S. Code of Federal Regulations (CFR) applicable to clinical studies (45 CFR 46 and 21 CFR including parts 50 and 56 concerning informed consent and IRB regulations); and completion of Human Subjects Protection Training.

The study informed consent documents will embody the elements of consent as described in the Declaration of Helsinki and International Conference on Harmonization Good Clinical Practice. Compliance with these standards provides public assurance that the rights, safety, and well-being of study subjects are protected, consistent with the principles that have their origin in the Declaration of Helsinki.

All key personnel (individuals responsible for the design and conduct of this study) will have completed Human Subjects Protection Training before interaction with any participants or to having access to their confidential study data.

Signature Page

The signatures below constitute the approval of this protocol and all attachments and provide the necessary assurances that this trial will be conducted according to all stipulations of the protocol, including all statements regarding confidentiality, and according to local legal and regulatory requirements and applicable guidelines.

Investigator Responsible for Participants:

Signed: ______________________________ Date: ______________

Dr Alphonse Ouedraogo, MD, PhD
Medical Officer, GRAS

Sponsor Investigator:

Signed: ______________________________ Date: ______________

Kathleen Neuzil, MD, MPH
Medical Officer, CVD

Table of Contents

Statement of Compliance ii
Signature Page iii
List of Abbreviations vi
Protocol Summary viii
1. Key Roles 1
2. Background Information and Scientific Rationale 3
2.1. Background and Significance 3
2.2. Rationale 6
2.3. Potential Risks and Benefits 7
3. Objectives 8
3.1. Study Objectives 8
3.2. Study Outcome Measures 9
4. Overview of Study Design 9
5. Enrollment and Withdrawal 10
5.1. Subjects 10
5.2. Subject Withdrawal 10
5.3. Termination of the Trial 11
6. Study Vaccine 11
6.1. TYPHIBEV® 11
6.2. Acquisition of Study Vaccine 11
6.3. Packaging and Labelling 11
6.4. Dosage, Preparation, and Administration of Study Vaccines 11
6.5. Accountability Procedures for the Study Vaccine 12
6.6. Concomitant Medications and Vaccinations 12
7. Study Schedule 12
7.1. Recruitment and Screening 12
7.2. Visit 1-Enrollment, Venipuncture, and Vaccination (Study Day 0) 12
7.3. Phone Call Follow-Up (Study Day 7) 13
7.4. Visit 2-Follow-Up, Venipuncture (Study Day 28) 13
8. Study Evaluations 13

8.1. Laboratory Evaluations 13
8.2. Reporting Procedures 13
9. Monitoring 14
9.1. Monitoring Plan 14
9.2. Inspections 14
9.3. Archiving 15
10. Statistical Considerations 15
10.1. Definition of Analysis Sets 15
10.2. Analysis Plan 15
11. Quality Control and Quality Assurance 15
11.1. General 15
11.2. Trainings 16
12. Ethics and Human Subjects Protections 16
12.1. Ethics Standards 16
12.2. Institutional Review Boards 16
12.3. Informed Consent Process 16
12.4. Subject Confidentiality 17
12.5. Study Discontinuation 17
12.6. Sharing Study Results 17
12.7. Use of Stored Specimens 17
12.8. Compensation 17
13. Data Handling and Record Keeping 17
13.1. Source Data and Access to Data 17
13.2. Data Recording and Record Keeping 18
13.3. Procedures for Reporting Protocol Deviations 18
14. Publication Policy 18
15. Timeframe and Duration of the Study 18
15.1. Timeline 18
15.2. Gantt Chart 19
References 19

List of Abbreviations

˚C	Degrees Celsius
AE	Adverse Event
BMGF	Bill & Melinda Gates Foundation
CERS	Comité d'Éthique pour la Recherche en Santé
CFR	U.S. Code of Federal Regulations
CI	Confidence Interval
CRF	Case Report Form
CVD	Center for Vaccine Development and Global Health
DU	D-antigen Unit
ELISA	Enzyme-Linked Immunosorbent Assay
EPI	Expanded Programme on Immunization
GCP	Good Clinical Practice
Gavi	Gavi, the Vaccine Alliance
GMC	Geometric Mean Concentration
GMT	Geometric Mean Titer
GRAS	Groupe de Recherche Action en Santé
HSA	Health Surveillance Assistant
ICF	Informed Consent Form
ICH	International Conference on Harmonisation
IDR	Incidence Density Ratio
IgA	Immunoglobulin A
IgG	Immunoglobulin G
IgM	Immunoglobulin M
IND	Investigational New Device
IPV	Inactivated Poliovirus Vaccine
IRB	Institutional Review Board
ISM	Independent Safety Monitor
LPS	Lipopolysaccharide
MAV	Meningitis A Vaccine
mIU/mL	Milli-International Units per Milliliter
MR	Measles Rubella Vaccine
MUAC	Middle Upper Arm Circumference
µg	Micrograms
mg	Milligrams
mL	Milliliters
NIH	National Institutes of Health
PI	Principal Investigator
PS	Polysaccharide

S. Typhi	*Salmonella enterica* serovar Typhi
SAE	Serious Adverse Event
SAGE	Strategic Advisory Group of Experts
SOP	Standard Operating Procedure
SMP	Site Monitoring Plan
STRATAA	Strategic Alliance on Typhoid across Africa and Asia
SUSAR	Suspected Unexpected Serious Adverse Reaction
TCV	Typhoid Conjugate Vaccine
TSAP	Typhoid Surveillance in Africa Program
TT	Tetanus Toxoid
TyVAC	Typhoid Vaccine Acceleration Consortium
VE	Vaccine Efficacy
Vi	Virulence
Vi-CRM	Vi-Typhoid Conjugate Vaccine conjugated to a variant of diphtheria toxin also known as Cross Reacting Material 197 (CRM197)
Vi-PS	Vi-Polysaccharide
Vi-rEPA	Vi conjugated to exoprotein A from Pseudomonas aeruginosa
Vi-TT	Vi-Typhoid Conjugate Vaccine conjugated to tetanus toxoid
YFV	Yellow Fever Vaccine

Protocol Summary

**Title:**	Immunogenicity and Tolerability of Typhoid Conjugate Vaccine (TCV) 5-6 Years After Routine Vaccines Administered With or Without TCV among Children Younger than 2 Years in Ouagadougou, Burkina Faso
**Participant Population:**	Up to 250 healthy children in Ouagadougou, Burkina Faso
**Number of Sites:**	Study is single center
**Participation Duration:**	28 days
**Description of Agent or Intervention:**	One 0.5 mL dose of Vi-CRM (TYPHIBEV®, Biological E, Hyderabad, India) at 5-6 years following their initial study vaccine dose (Vi-TT [Typbar TCV®], Bharat Biotech, Hyderabad, India or IPV).
**Objectives:**	1. Determine typhoid-specific antibody responses approximately 5-6 years after an initial dose of Vi-TT given at 9-23 months of age.<br>2. Determine typhoid-specific antibody responses to a dose of Vi-CRM given 5-6 years after a dose of Vi-TT given at 9-23 months of age.<br>3. Determine typhoid-specific antibody responses to a first dose of Vi-CRM given at approximately 6-7 years of age.<br>4. Determine safety profile of Vi-CRM given 5-6 years after Vi-TT at 9-23 months of age.
**Description of Study Design:**	This study will include children who participated in the previous Phase 2 Randomized, Double-Blind, Controlled Trial of the Safety and Immunogenicity of Typhoid Conjugate Vaccine (Vi-TT) among Children Younger than 2 Years in Ouagadougou, Burkina Faso. Children will be contacted and asked to return to the study clinic for either a first or second TCV dose. For the previous Phase 2 study, a TCV using tetanus toxoid (TT) as the carrier protein was used. For this study, a TCV using a diphtheria toxin variant CRM197 (CRM) will be used. Both vaccines are prequalified by the World Health Organization (WHO). Solicited adverse events (AE) will be collected for 7 days after vaccination, and unsolicited AEs will be collected for 28 days after vaccination. Blood will be drawn at enrollment and 28 days after for immunogenicity assessments. The purpose of this blood draw is to assess the

	immune responses to Vi-CRM. Serum specimens will be collected to quantify anti-Vi antibodies.
**Inclusion and Exclusion Criteria:**	**Inclusion Criteria:** Each participant must satisfy the following inclusion criteria:<br>• Male or female child 9 months through 23 months of age at the time of enrollment into the completed Phase 2 vaccine trial.<br>• A child whose parent or guardian has voluntarily given informed consent.<br>**Exclusion Criteria:** No participant may have any of the following exclusion criteria at study entry:<br>• Receipt of blood products in the last 6 months.<br>• Prior receipt of TCV outside of the study as part of a vaccination campaign or through routine vaccination<br>• Any condition determined by the investigator likely to interfere with evaluation of the vaccine or to be a significant potential health risk to the child.
**Estimated Time to Complete Enrollment:**	Full enrollment is anticipated over approximately 2 months.

Table 1. Study Schedule of Events

**Study Visit Type**	**Recruitment and Screening**	**Eligibility, enrollment, vaccination**	**Phone call for solicited adverse events**	**Final visit**
**Study Visit Number**	**V00**	**V01**	**P01**	**V02**
**Study Time point**		**D0**	**D7**	**D28**
Study introduction, recruiting	X	X		
Obtain informed consent	X	X		
Review eligibility criteria	X	X		
Record solicited adverse events			X	
Record unsolicited adverse events		X	X	X
3-5 mL venous blood collection for immunogenicity		X		X

1. Key Roles

Individuals

Sponsor Investigator:

Kathleen Neuzil, M.D.
Director, Center for Vaccine Development and Global Health (CVD)
University of Maryland School of Medicine
685 West Baltimore St, Rm 480
Baltimore, MD 21201
Phone: +1 410 706-3502
Fax: +1 410 706-6205
Email: kneuzil@som.umaryland.edu

Investigator Responsible for Participants:

Alphonse Ouedraogo, MD, PhD
Director, Groupe de Recherche Action en Santé (GRAS)
06 BP 10248 Ouagadougou 06 BURKINA FASO
Phone: (+226) 25 35 56 90
Cel: (+226) 70 20 04 44/76 20 04 44/78 20 04 44
Fax: (+226) 25 35 56 73
Email: gras@fasonet.bf
Back-up E-mail: a.ouedraogo@gras.bf

Co- investigators

Sodiomon B. Sirima, MD, PhD
Groupe de Recherche Action en Santé (GRAS)
06 BP 10248 Ouagadougou 06 BURKINA FASO
Phone.(+226) 25 35 56 90
Fax: (+226) 25 35 56 73

Alfred B. Tiono MD, PhD
Groupe de Recherche Action en Santé (GRAS)
06 BP 10248 Ouagadougou 06 BURKINA FASO
Phone.(+226) 25 35 56 90
Fax: (+226) 25 35 56 73

Issa Nébié Ouédraogo MsC, PhD
Groupe de Recherche Action en Santé (GRAS)
06 BP 10248 Ouagadougou 06 BURKINA FASO
Phone.(+226) 25 35 56 90

Fax: (+226) 25 35 56 73

Matthew Laurens, MD, MPH
CVD, University of Maryland School of Medicine
685 West Baltimore St, Rm 480
Baltimore, MD 21201
Phone: +1 410 706-5328
Fax: +1 410 706-6205
Email: mlaurens@som.umaryland.edu

Institutions

Sponsor:

Center for Vaccine Development and Global Health (CVD)
University of Maryland School of Medicine
685 West Baltimore St, Rm 480
Baltimore, MD 21201
Contact: Kathleen Neuzil, MD
Phone: +1 410 706-3502
Fax: +1 410 706-6205
Email: kneuzil@som.umaryland.edu

Funding Agency:

Center for Vaccine Development and Global Health (CVD)
University of Maryland School of Medicine
685 West Baltimore St, Rm 480
Baltimore, MD 21201

Institutional Review Boards:

University of Maryland Institutional Review Board (IRB)
620 W. Lexington St
Baltimore, MD 21201
phone: +1 410 706-5037
email: hrpo@umaryland.edu

Comité d'Éthique pour la Recherche en Santé (CERS)
Ouagadougou, Burkina Faso

2. Background Information and Scientific Rationale

2.1. Background and Significance

2.1.1. Introduction and Literature Review

Typhoid fever is an acute infection with *Salmonella enterica* serovar Typhi (*S.* Typhi) acquired through exposure to food or water contaminated with human feces. Humans are the sole reservoir of *S*. Typhi. In endemic areas, the incidence of typhoid fever has traditionally been highest in school-age children (5-19 years) and is being increasingly recognized in the under-5-year age group. In countries with inadequate sanitation and water contamination, infections may be acquired from water and food contaminated with human waste. In addition, typhoid may be transmitted via a food vehicle handled by an individual who is a chronic carrier of *S*. Typhi.

An estimated 9 million cases of typhoid fever and 110,000 related deaths occur worldwide, each year.[1,2] However, it should be recognized that estimating typhoid fever incidence is challenging in low- and middle-income countries due to the non-specific clinical presentation of the disease, which mimics many other common clinical syndromes (e.g., malaria), and limited access to blood culture, the most reliable laboratory-based diagnostics.

Typhoid fever may be asymptomatic or present clinically with mild to severe disease. Clinical disease includes high fever, headache, rash, abdominal pain, malaise, myalgia, and anorexia. Serious complications of typhoid fever include intestinal perforation and hemorrhage, or neurological complications; this is primarily observed in those whose illness has been untreated for longer than 2 weeks. An estimated 2-5% of patients become chronic carriers of *S*. Typhi, with the bacteria colonizing the gall bladder of affected individuals with persistent shedding of the bacteria in the stool.

2.1.2. Burden of Typhoid Fever

2.1.2.1. Typhoid Fever in Africa

The epidemiology of typhoid fever in Africa has been less well studied than in Asia, although the disease is endemic on both continents. The Typhoid Fever Surveillance in Africa Program (TSAP) measured the incidence of invasive *Salmonella* bloodstream infections across 13 sites, in 10 countries in Africa, over a 27-month period.[3] A major finding was that the overall adjusted incidence of typhoid fever was 2-3 times higher than previous estimates.[4] The overall annual incidence was 10-100 cases/100,000 people, comparable to cases reported in Asia. However, there was marked heterogeneity across the continent, even within countries such as Ghana, Burkina Faso, and Kenya. Incidence was highest in children 2-14 years old. Importantly, 47% of *S*. Typhi isolates were multidrug-resistant.

In sub-Saharan Africa, the annual mortality rate from typhoid fever is 3.3/100,000 people, accounting for 250 years of healthy life lost/100,000 people. The mortality rate is highest for boys and girls aged 1-4 years. Outbreaks of antibiotic-resistant typhoid fever have occurred throughout sub-Saharan Africa in recent years, including in Malawi, Uganda, Zimbabwe, Zambia, and the Democratic Republic of Congo.[5-9]

2.1.2.2. Typhoid Fever in Burkina Faso

In 2009, the International Vaccine Institute (IVI) began TSAP and implemented standardized surveillance of typhoid fever across sub-Saharan Africa. Data from TSAP show the adjusted incidence of *S*. Typhi ranged from 104 to 383/100,000 person years of observation (PYO)[3] for all ages across the two study sites in Burkina Faso. By age group, the adjusted incidence in Nioko II, one of the Burkina Faso sites, was highest for children 5-14 years (315/100,000 PYO), whereas at the Polesgo site, the highest adjusted incidence occurred in children 2-4 years of age (1890/100,000 PYO).[3]

2.1.3. Prevention of Typhoid Fever with Vaccines

2.1.3.1. Previous Vaccines

Before 2017, two typhoid vaccines were available[10]: 1) oral, live attenuated Ty21a vaccine licensed for children six years of age and older, and 2) an injectable Vi capsular polysaccharide vaccine licensed for children two years of age and older. These vaccines are underutilized in high-burden countries despite typhoid's substantial and detrimental impact and World Health Organization (WHO) recommendation for their use. It has been difficult to incorporate these vaccines into routine immunization programs in low-resource countries because neither is approved for children younger than two years of age or financed by Gavi, the Vaccine Alliance (Gavi).

Polysaccharide vaccines, such as those developed against *Neisseria meningitidis, Streptococcus pneumoniae, Haemophilus influenzae* type b (Hib), and *S.* Typhi prevent infection by inducing an immune response against specific capsular polysaccharides. Like other pure polysaccharide vaccines, Vi polysaccharide vaccine does not induce immunologic memory and is poorly immunogenic in young children under two years of age. These deficiencies have been addressed by conjugating Vi to a carrier protein. A TCV conjugated with a recombinant exoprotein A from *Pseudomonas aeruginosa* (rEPA) was highly efficacious in Vietnamese children[11], but never made it to licensure.

2.1.3.2. Typbar Typhoid Conjugate Vaccine

A typhoid Vi conjugate vaccine (Vi-TT, Typbar-TCV®) has been developed by Bharat Biotech International, Hyderabad, India. This vaccine consists of 25 µg of Vi polysaccharide conjugated to a nontoxic TT protein carrier. Because the polysaccharide antigen is conjugated to an immunogenic protein, Vi-TT stimulates a T cell-dependent response that results in immune responses in young children and immunologic memory. Vi-TT elicits a stronger anti-Vi response than unconjugated Vi polysaccharide and the elicited antibodies have higher avidity than those detected after the unconjugated Vi vaccine.

The safety and immunogenicity of Vi-TT has been studied in adults and children as young as six months of age in India, a typhoid endemic area.[12] Vi-TT given as a single dose via the intramuscular route was well tolerated and induced strong serum anti-Vi IgG responses in children at least six months of age and in adults.[12] In an adult volunteer challenge study at the University

of Oxford, a single dose of Vi-TT was well tolerated and induced 54.6% protective efficacy for the conjugate (and 52.0% efficacy for the Vi polysaccharide alone) at 28 days after vaccination. Post-hoc analyses of alternative clinical diagnosis criteria for typhoid fever showed Vi-TT was 87.1% effective against the outcome of fever, of at least 38.0˚C, with subsequent bacteremia.[13,14]

In October 2017, the WHO Strategic Advisory Group of Experts (SAGE), in recognition of the continued high burden of typhoid and increase in antimicrobial resistance of *S*. Typhi in low- and middle-income countries, recommended the introduction of TCVs for children six months and older in typhoid endemic countries.[15] Following the SAGE recommendation, Gavi announced an 85 million dollar funding window for 2019-2020 to support the introduction of TCVs in low-income countries.[16] In December 2017, WHO approved the prequalification of Typbar-TCV, facilitating its introduction in low-income countries.[17]

SAGE also encouraged studies of co-administration with routine Expanded Programme on Immunization (EPI) vaccines in the first two years of life. The first study to administer Vi-TT at the 15-month EPI visit with measles-rubella (MR) and meningitis A vaccine was conducted among children ages 15 to 23 months in Ouagadougou, Burkina Faso.[18] Children were recruited and vaccinated from December 3, 2018 to February 18, 2019. The vaccine was well-tolerated and generated robust immunity without interference with group A meningococcal conjugate vaccine and MR. Immunogenicity data was generated at 28 days and 6 months after immunization.

In summary, this Vi conjugate vaccine is a promising candidate for typhoid fever control in Africa because of its one-dose schedule, demonstrated immunogenicity, and safety profile in young children.

2.1.3.3. TYPHIBEV® Typhoid Conjugate Vaccine

A second typhoid Vi conjugate vaccine (TYPHIBEV®) has been developed by Biological E., Ltd., Hyderabad, India. This vaccine, Vi-CRM, consists of 25 µg of Vi polysaccharide from Citrobacter freundii, which is clinically indistinguishable from the Vi polysaccharide from *S*. Typhi, conjugated to a variant of diphtheria toxin (CRM197) as the carrier protein. Conjugation transforms the antigen into a T-cell dependent antigen, capable of inducing immunological memory and an immune response in infants and young children.

The safety and immunogenicity of Vi-CRM has been studied in a Phase 1/2 clinical trial in adults 18-40 years of age in Belgium.[19] Vaccines were well-tolerated, and no safety signal was detected. Compared to licensed polysaccharide-based vaccines, Vi-CRM formulations induced higher antibody concentrations. Anti-Vi-CRM antibody responses were dose dependent, such that among doses studied (1.25 µg, 5.0 µg, 12.5 µg, and 25 µg), the 25 µg dose elicited the highest antibody level and at least 95% seroconversion at four weeks post-vaccination. Ninety-five percent of participants receiving the 25 µg dose achieved 95% seroconversion at 6 months post-vaccination.

In a randomized comparative licensure study,[19,20] 622 healthy subjects were enrolled at nine clinical sites into one of two treatment arms. A total of 311 subjects received a single 0.5 mL dose

of either Vi-CRM or a comparator vaccine. All subjects were further stratified into three age subsets as follows: 1) >6 months to <2 years, 2) <2 years to 18 years, and 3) >18 to 64 years of age. The most frequently reported local AEs after administration of Vi-CRM were injection site pain (4.50%), injection site erythema (0.64%) and injection site swelling (0.32%). These usually occurred within the first 48 hours and resolved within 2-3 days. The most frequently reported systemic AEs were pyrexia (2.25%), vomiting (0.96%), headache (0.64%), fatigue (0.64%), arthralgia (0.32%), and rash (0.32%). No vaccine-related serious AEs (SAEs) were reported. Overall immune responses to the vaccine included anti-Vi IgG antibody concentrations above the short-term protective seroconversion threshold value of >2.0μg/mL and above the WHO-defined protective seroconversion threshold value of >4.3μg/mL were 98.98% and 95.59%, respectively. Seroconversion rated in >6 months to 2 years, <2 years to 18 years, and >18 to 64 years of age were 99.22%, 100.0%, and 97.62% using the short-term threshold value of >2.0μg/mL and 96.90%, 95.12%, and 94.05% using the WHO defined threshold value of >4.3μg/mL, respectively. The overall proportion of subjects achieving >4-fold increase in anti-Vi IgG antibody concentrations 42 days after vaccination was 96.95%.

The Vi-CRM vaccine was prequalified by WHO in December 2020, meeting stringent international standards for purchase by United Nations agencies.

In summary, this Vi-CRM vaccine is another promising tool for the control of typhoid fever in Africa because of its favorable safety profile, one-dose schedule, and licensure down to 6 months of age. The WHO views the Vi-TT and Vi-CRM vaccines as interchangeable from a policy perspective.

2.2. Rationale

Clinical studies of TCV conducted with TyVAC funding have complemented and extended the data generated by the manufacturers for licensure, and have demonstrated the safety, efficacy, and immunogenicity of different schedules of TCV in diverse low-resource pediatric populations.[2,18,21-28] Currently, the duration of protection of TCVs and the need and timing of booster doses, are critical public health questions. SAGE will be reconvening the Typhoid Vaccine Working Group in Spring 2024 to begin to address these questions, with a tentative SAGE review date of Spring 2025.

The Malawi randomized controlled efficacy trial (RCT) demonstrated that a single dose TCV is approximately 80% efficacious against blood culture-confirmed typhoid fever for at least four years.[2] However, children who receive TCV at younger than two years of age have a lower point estimate of efficacy at four years and have a steeper decline of antibody responses over time. While neither of these trends are statistically significant, they do raise the question of whether a booster dose of TCV will be needed to protect vaccinees, particularly those who receive TCV in infancy, through childhood. Further, unpublished data from the Bangladesh cluster-randomized trial (CRT) demonstrated a faster waning of protection than was demonstrated in the Malawi RCT. While not a placebo-controlled study, the Bangladesh analysis compared cohorts in the original CRT who were randomized to receive TCV to those who received TCV approximately two years later when the study was unblinded.

In Malawi, the 9-11-month-old cohort of children in the RCT provided a unique opportunity to evaluate a booster dose of TCV at 5 years of age. We demonstrated that a second dose of Vi-TT administered at age 5 years, after a first dose of Vi-TT at 9-11 months of age, was well-tolerated and produced a significantly more robust immune response than a first dose of Vi-TT at 5 years of age (Figure 1). That trial was originally intended to include two booster groups, one with Vi-TT and one with Vi-CRM, but unfortunately the companies would not agree to a head-to-head comparison. Therefore, we have no data on the tolerability or immunogenicity of a booster response with a heterologous TCV.

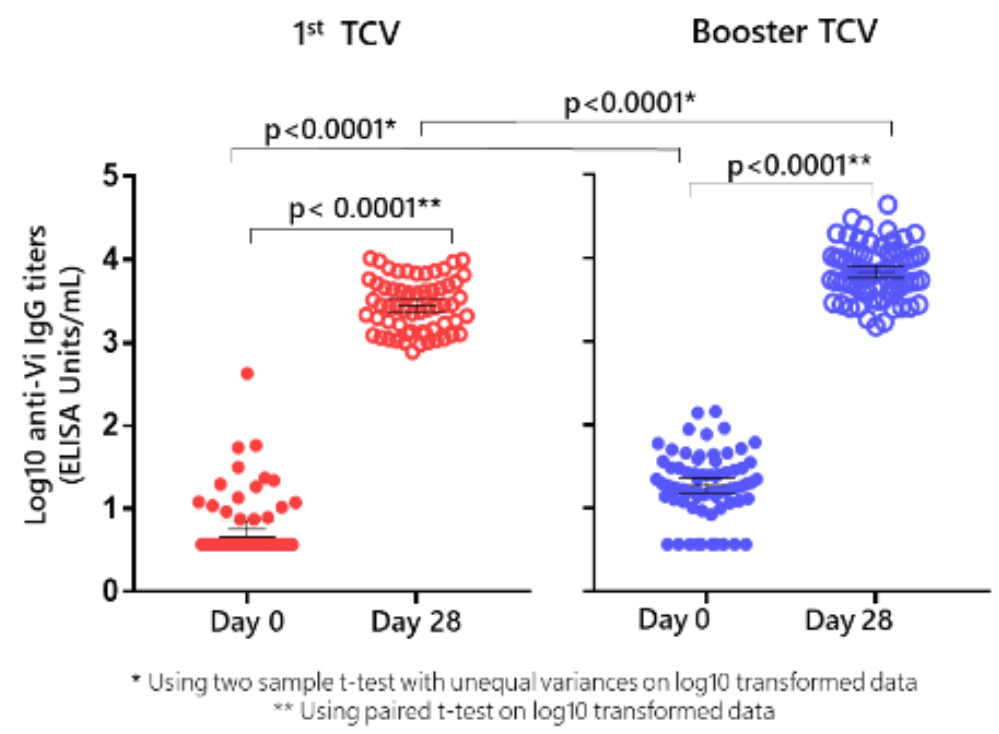

Figure 1. Anti-Vi IgG geometric mean titer before and 28 days after Vi-TT 4+ years after first TCV or Men-A.

In an RCT in Burkina Faso, we had previously demonstrated the immunogenicity of TCV when co-administered with routine EPI vaccines in two cohorts, at 9-months[26] and 15-months.[18] These children are now about 5 years past their first dose of vaccine. Thus, they provide a unique opportunity to evaluate the reactogenicity and immunogenicity of a dose of a heterologous TCV (Vi-CRM) given at a primary school time point, approximately 5-6 years after the first dose of Vi-TT and compare to a first dose of Vi-CRM given at the same primary school time point. These data will be highly relevant to public health decision-making.

2.3. Potential Risks and Benefits

2.3.1. Potential Risks

2.3.1.1. Potential Risks of TCV

Like any vaccine, Vi-CRM may cause side effects in some children. The safety of Vi-CRM has been assessed in clinical trials.[19,20] In general, the vaccine was well-tolerated. Common side effects are similar in nature and frequency to those caused by other vaccines. The most frequently reported local side effects after administration of Vi-CRM were injection site pain (4.50%), injection site erythema (0.64%) and injection site swelling (0.32%).[20] These usually occurred within the first 48 hours and resolved within 2-3 days. The most frequently reported systemic AEs were pyrexia (2.25%), vomiting (0.96%), headache (0.64%), fatigue (0.64%), arthralgia (0.32%), and rash (0.32%).[20] All of these side effects were transient and no AEs leading to withdrawal from clinical trial have been reported. No vaccine-related SAEs were reported.

Trained clinical staff will be on-site during immunizations, conducted at premises equipped for any immediate and/or emergency care that may be needed. If Vi-CRM causes an immediate reaction or unknown side effect, the participant will be assessed and treated at no cost to the participant or his/her family.

2.3.1.2. Potential Risks to Participant Privacy

To ensure follow-up of the correct study participant, personal identifiers, including birth date, sex, will be collected and recorded on electronic data collection forms. As a result, a potential risk for “loss of confidentiality” exists. To minimize this risk, participants will be assigned a unique study participant number that will be used to identify the participant and link, using a master linking document, an individual to his/her study data and/or biological specimens. Whenever feasible, use of identifiers will be avoided, and the unique study participant numbers will be used instead. Names and addresses will only be viewable by study staff, the Sponsor, and representatives of the Sponsor when reviewing study data onsite. These identifiers will not be included in study information sent off-site, including copies of the study data and reports/publications. Paper-based records will be kept in a secure location and accessible only to personnel involved in the study. Computer-based files will only be made available to personnel involved in the study through the use of access privileges and passwords. Individual participants will not be identified in any study-related reports, and all study results will be reported in aggregate only.

Biological specimens will be identified by study participant number and specimen collection date only. The specimens will be stored at the GRAS laboratory, where they will be processed and stored according to standard operating procedures (SOPs). Aliquots from children will be shipped, tested and stored at the University of Maryland CVD for antibody testing in accordance with international air transport authority regulations.[29] When stored at GRAS or CVD, the specimens will be under the responsibility of the investigator. The links between study data and the study specimens will not be destroyed, but no personal-identifying information associated with the study will be stored with the specimens.

2.3.2. Known Potential Benefits

All participants enrolled will have the benefit of receiving Vi-CRM vaccine. The Vi-CRM vaccine provides protection against typhoid fever. There is no routine vaccination program for typhoid in Burkina Faso, though this is anticipated in the near future.

The trial will help guide future implementation of TCV vaccination programs by informing the safety and immune responses to a first or second dose of TCV in young children at school entry. Whilst participants would not necessarily benefit directly from this, results from this trial could lead to implementation of a two-dose TCV vaccine program in typhoid endemic areas, providing extended direct and indirect protection to both those close to the participant and those in the wider population.

3. Objectives

3.1. Study Objectives

- 3.1.1. Determine typhoid-specific antibody responses approximately 5-6 years after an initial dose of Vi-TT given at 9-23 months of age.
- 3.1.2. Determine typhoid-specific antibody responses to a dose of Vi-CRM given 5-6 years after a dose of Vi-TT given at 9-23 months of age.
- 3.1.3. Determine typhoid-specific antibody responses to a first dose of Vi-CRM given at approximately 6-7 years of age.

3.1.4. Determine safety profile of Vi-CRM given 5-6 years after Vi-TT at 9-23 months of age.

3.2. Study Outcome Measures

3.2.1. Typhoid-specific antibody responses, measured by ELISA for anti-Vi percent seroconversion (>4-fold increase in IgG-specific (or IgA-specific) anti-Vi antibody titers) and geometric mean titer (GMT).
3.2.2. Proportion of participants who develop solicited AEs detected in the 7 days after vaccination.
3.2.3. Proportion of participants who experience SAEs up to 28 days after vaccination.
3.2.4. Proportion of participants who experience other non-serious AEs up to 28 days after vaccination.

4. Overview of Study Design

This study will comprise children who were initially vaccinated with TCV or control vaccine at 9 through 23 months of age. As these participants were previously randomized to receive either TCV (Vi-TT) or control vaccine (inactivated polio vaccine, IPV) initially, those who already received TCV will be receiving a second dose of TCV (Vi-CRM), and those who received IPV will be receiving a first dose of TCV (Vi-CRM). The purpose of this detailed evaluation is to compare the immunogenicity of a first dose of TCV given at age 6-7 years of age to the immunogenicity of a second dose of TCV given at 6-7 years of age to children receiving primary dose at 9-24 months of age. Serum specimens will be collected on day 0 (before vaccine administration) and at 28 days after vaccination from all children in the study. These children will have antibody to typhoid assessed at baseline (day 0) and 28 days after vaccination. All children will be assessed at 7 days after vaccination for solicitation of local and systemic AEs. AEs will be assessed up to 28 days after vaccination administration.

In summary, this Vi-CRM vaccine is another promising tool for the control of typhoid fever in Africa because of its one-dose schedule and its demonstrated immunogenicity and safety profile in infants and children. At this time, the need and timing for a booster dose is unknown. In Burkina Faso and other countries, a single dose TCV will be administered through routine EPI at the 9-month vaccination visit. This study will assess the reactogenicity and immunogenicity of a second dose of TCV given at a primary school time point, approximately 5-6 years after the first dose, and compare to a first dose of vaccine given at the same primary school time point.

Children who participated in the Phase 2 study and were enrolled and vaccinated in 2018-2019 will be eligible. Serum specimens will be collected and tested for the presence of serum IgG and IgA anti-Vi antibody. The prior TCV vaccination status of these children will remain blinded to study staff and laboratory technicians, and it will only be known by the study biostatistician.

5. Enrollment and Withdrawal

5.1. Subjects

Following community sensitization using IRB-approved materials, local community families whose children participated in the previous TCV vaccine study and were 9 through 23 months of age at vaccination will be contacted and given information about the study either individually or in a group setting. This information will be given orally and in writing. The parents of eligible children will be given an opportunity to ask questions and provide written informed consent in a semi-private area before enrollment and venipuncture.

5.1.1. Inclusion Criteria

Each participant must satisfy all of the following inclusion criteria:

- Male or female child 9 months through 23 months of age at the time of enrollment into the completed Phase 2 vaccine trial.
- A child whose parent or guardian has voluntarily given informed consent.

5.1.2. Exclusion Criteria

No participant may have any of the following exclusion criteria at study entry:

- Receipt of blood products in the last 6 months.
- Prior receipt of TCV outside of the study as part of a vaccination campaign or through routine vaccination
- Any condition determined by the investigator likely to interfere with evaluation of the vaccine or to be a significant potential health risk to the child.

5.1.3. Temporary Exclusion Criteria

The following will be considered temporary contraindications to enrollment and vaccination. If these apply, the participant will be temporarily excluded for vaccination until 48 hours has passed. A re-assessment will be needed to ensure these temporary exclusion criteria no longer exist.

- Reported fever within 24 hours prior to vaccination.
- Use of anti-pyretics within 4 hours prior to vaccination.

5.2. Subject Withdrawal

5.2.1. Reasons for Withdrawal

Participants' parents/guardian may withdraw consent at any point.

The Investigator may also discontinue a participant from the trial at any time if the Investigator considers it necessary for any reason including:

- Ineligibility (either arising during the trial or retrospectively having been overlooked at screening),
- Significant protocol deviation,
- Significant non-compliance with trial requirements,

- An AE or disease progression resulting in the inability to continue to comply with trial procedures and follow-up.

5.2.2. Handling of Withdrawals

Withdrawal will result in cessation of any blood tests. Data and blood samples collected before the time of withdrawal will be kept and analyzed as part of the study data. Reasons for withdrawal from the study, if known, will be recorded in the participant's case report form (CRF).

5.3. Termination of the Trial

5.3.1. Termination According to the Protocol

The study will end once all participants have undergone venipuncture.

5.3.2. Suspension and/or Premature Termination

The regulatory authorities and study sponsor(s) retain the authority to suspend additional enrollment and study interventions for the entire study, as applicable.

6. Study Vaccine

6.1. TYPHIBEV®

TYPHIBEV® (Vi-CRM) consists of 25 µg of Vi polysaccharide conjugated to a protein carrier that is a variant of diphtheria toxin (CRM197). Vi-CRM is given as a 0.5-ml dose by the intramuscular route to infants, toddlers, children, and adults. This vaccine will be shipped from the manufacturer in single-dose (0.5 mL) or five-dose (2.5 mL) vials.

6.2. Acquisition of Study Vaccine

Biological E., Ltd. (Hyderabad, India) will donate Vi-CRM vaccine.

6.3. Packaging and Labelling

6.3.1. Packaging

TYPHIBEV® is packaged in a single-dose (0.5 mL) vial or a five-dose (2.5 mL) vial.

6.3.2. Labelling

The vaccines will be labelled by the manufacturer and will not be relabeled.

6.3.3. Storage and Stability

Vi-CRM will be stored at 2-8˚C in a temperature monitored refrigerator. Opened, multidose vials from which one or more doses of vaccine have been removed during an immunization session may be used in subsequent immunization sessions for 28 days, provided that the expiry date has not passed, the vaccine is stored under appropriate cold chain conditions, the vaccine vial septum has not been submerged in water, aseptic technique has been used to withdraw all doses, and the vaccine vial monitor has not reached the discard point.[20] Refrigeration temperature and cold chain will be monitored during the study.

6.4. Dosage, Preparation, and Administration of Study Vaccines

6.4.1. Dosage

The appropriate dosage of Vi-CRM is 0.5 mL, which includes 25 µg purified Vi capsular polysaccharide of *S.* Typhi conjugated to diphtheria toxin (Vi-CRM).

6.4.2. Preparation

Vi-CRM is a Vi polysaccharide-diphtheria toxin conjugate vaccine that is manufactured using Citrobacter freundii sensu lato 3056 (Vi) and is conjugated to a variant diphtheria toxin (CRM197) as a carrier protein (25 µg/dose). Sodium chloride (q.s.) and phosphate buffer (q.s.) are used as a diluent (buffering agent) in vaccine formulation. 2-Phenoxyethanol (5 mg/dose) is used as a preservative. The vaccine is packaged as a single-dose (0.5 mL) or five-dose (2.5 mL) vial.

6.4.3. Administration

Vi-CRM should be administered as an intramuscular injection to the upper arm.

6.5. Accountability Procedures for the Study Vaccine

The vaccines will be cold shipped to Burkina Faso and cleared by GRAS staff and agents. They will then be transported to GRAS headquarters and distributed to the vaccination site while maintaining the cold-chain (aiming for temperature between 2-8˚C). The number of doses of study vaccines that are received, used, and wasted will be documented daily during the study, and checked weekly. Unused vaccines at the end of the trial may be retained for donation to the Burkina Faso EPI, or destroyed if this is not feasible or if vaccines are beyond expiration. Any recall of study vaccines required for use in the study or reporting of defective vaccines will be performed according to trial SOPs.

6.6. Concomitant Medications and Vaccinations

Concomitant medications and vaccinations will be checked and recorded on CRFs.

7. Study Schedule

7.1. Recruitment and Screening

Parents of children who participated in the previous TCV vaccine trial will be given general information about the current study and the eligibility criteria. The study will be explained, and the requirements discussed. (See Section 12.3 for more details on informed consent). Study staff will answer any questions about the study, procedures, benefits, and risks. The parents/guardians of the children will then have the opportunity to provide informed consent. After informed consent is obtained, each potential participant will be screened for eligibility according to the inclusion/exclusion criteria. Those who are eligible may proceed to enrollment.

7.2. Visit 1-Enrollment, Venipuncture, and Vaccination (Study Day 0)

Screening and enrollment visits may occur on the same day. At the enrollment visit, and before venipuncture or vaccination, informed consent will be confirmed. Temperature will be taken, and concomitant medications recorded for all participants. Study staff will review eligibility criteria with all participants and their parent/guardian. All participants will then have 3-5 mL of blood drawn for immunogenicity testing. Once this is complete, participants will receive Vi-CRM.

7.3. Phone Call Follow-Up (Study Day 7)

Study staff will contact each participant's parent/guardian via cell phone to collect solicited and unsolicited AEs and document any concomitant medications.

7.4. Visit 2-Follow-Up, Venipuncture (Study Day 28)

Participants will return to clinic 28 days after vaccination. Study staff will document any AEs and record any concomitant medications. All participants will have 3-5 mL of blood drawn for immunogenicity testing. This is the final study visit.

8. Study Evaluations

8.1. Laboratory Evaluations

8.1.1. Clinical Laboratory Evaluations

Laboratory assays will be used for research purposes to assess the immunogenicity of Vi-CRM.

8.1.2. Blood Specimens

8.1.2.1. Collection of Clinical Specimens

All participants will have approximately 3-5 mL of blood drawn to assess vaccine immunogenicity as described above.

8.1.2.2. Conditions for Handling, Transport, and Storage of Specimens

Blood samples taken for the immunogenicity assessment will be transported to GRAS research laboratories daily where they will be processed and stored at -20˚C. Samples will be shipped to the University of Maryland laboratory in Baltimore, MD for antibody testing.

8.1.3. Assays

8.1.3.1. Anti-Vi IgG and IgA Antibody ELISA

The primary laboratory technique performed will be anti-Vi IgG and IgA antibody ELISA performed on the extracted plasma sample, using a commercially available assay (Vacczyme Binding Site, or another comparable assay). This assay will be performed according to the manufacturer's instructions at the University of Maryland laboratory in Baltimore, MD.

8.2. Reporting Procedures

8.2.1. Adverse Events

All AEs related to study procedures will be recorded, including a description of the event, date of onset and resolution, severity, assessment of relatedness to the study, and action taken.

8.2.2. Serious Adverse Events

Life-threatening SAEs or death must be reported via telephone or email to the Sponsor and the local regulatory organizations within 3 business days after notification of the event. All SAEs will be reported to the Institutional Review Board/Ethics Committee and the manufacturer according to requirements. Related and unexpected deaths will be reported within 24 hours.

9. Monitoring

9.1. Monitoring Plan

Monitoring will be conducted according to the sponsor's requirements. The individuals responsible for monitoring the study will periodically review the progress of the study and should have access to all records necessary to ensure the ethical and safety conduct of the study and the integrity/validity of the recorded data.

During site visits and contacts, the monitor will:

- Check and assess the progress of the study.
- Review study data collected.
- Perform source data verification.
- Review regulatory files.
- Identify any issues and address their resolution.

This will be done in order to verify that:

- The data are authentic, accurate and complete.
- The safety and rights of subjects are being protected.
- The study is conducted in accordance with the approved protocol (and any subsequent amendment) and all applicable regulatory requirements.

As part of study conduct, the principal investigator agrees to allow the monitor or sponsor representative direct access to all relevant documents and to allocate his/her time and the time of his/her staff to the monitor to discuss findings and any relevant issues.

9.1.1. Close-Out Visit

Upon completion of the study, the study sponsor will conduct the following activities:

- Data clarification and/or resolution
- Destruction of unused stored samples for which participants have not consented storage
- Review of site study records for completeness

9.2. Inspections

For the purpose of compliance with applicable regulatory guidelines it may be necessary for the sponsor or national or foreign regulatory authorities to conduct a site audit. This may occur at any time from start to after conclusion of the study.

The investigator agrees to allow the auditor direct access to all relevant documents and to allocate his/her time and the time of his/her staff to the auditor to discuss findings and any relevant issues.

National and foreign regulatory authorities may conduct a regulatory inspection of this study. If a regulatory authority requests an inspection, the investigator must inform the sponsor immediately about this request. The investigator agrees to allow the inspector(s) direct access to all relevant

documents and to allocate his/her time and the time of his/her staff to the inspector(s) to discuss findings and any relevant issues.

9.3. Archiving

The principal investigator will maintain all records pertaining to this study for at least 2 years following the date a marketing application is approved for the TYPHIBEV vaccine in Burkina Faso, or if no application is to be filed or if the application is not approved, until 5 years following completion of clinical study report.

10. Statistical Considerations

Children 9 through 23 months of age at the time of initial vaccination in 2018-2019 will be eligible. Participants (n=250) were initially randomized to receive Vi-TT (n=148) or IPV (102). With an expected 25% inability to contact/recruit rate, we will anticipate 187 children (75% of 250 enrolled) will complete follow-up assessments, 111 in the initial Vi-TT group and 77 in the initial IPV group. The study will analyze data in a descriptive manner.

10.1. Definition of Analysis Sets

10.1.1. Intention-to-Treat Population

The intention-to-treat (ITT) population includes all enrolled participants. The primary analysis will be based on this population.

10.1.2. Per-Protocol Population

The per-protocol (PP) population includes all vaccinated individuals who complete the study without any protocol deviations.

10.2. Analysis Plan

Details of study analyses will be included in a statistical analysis plan written by the study biostatistician before study data analysis begins.

11. Quality Control and Quality Assurance

11.1. General

The study will be conducted in accordance with the procedures specified in the protocol and staff will be guided by standard operating procedures (SOPs). Study data collection forms are designed to guide staff study conduct; forms also include areas for documenting that activities did occur (even if these activities did not require recording of data) and in the appropriate sequence. All study staff, including temporary staff such as nurses and interviewers, must attend mandatory training before participant enrollment.

Study-specific SOPs will be developed and documented for key study procedures and refined/revised, as necessary.

Site monitoring will be conducted to ensure that human subject protection procedures and study procedures, including biological specimen collection, are of high quality and that the study is conducted in accordance with the protocol.

After data have been entered in the study database, they will be checked systematically by data management staff according to a pre-specified data validation plan. All listings of the database will be reviewed and discussed for assessment of consistency and medical plausibility. After resolution of all issues, the Statistical Analysis Plan (SAP) will be finalized, and the database will be locked after resolution of any remaining queries. An audit trail will be kept of all subsequent changes to the data.

11.2. Trainings

Trainings for the research team (including the investigator, project manager/study coordinator, researchers, physicians, nurses, and technicians) participating in the clinical trial will include: the basics of research and ethics, information on how to conduct the trial, the standard operation procedures (SOPs) to be used in the trial.

12. Ethics and Human Subjects Protections

12.1. Ethics Standards

The study will be conducted in accordance with the principles of the Declaration of Helsinki and in accordance with principles of Good Clinical Practice.

12.2. Institutional Review Boards

This protocol, and informed consent form will be submitted to the University of Maryland Institutional Review Board and the Ethics Committee for Health Research in Burkina Faso for written approval. Reports will be provided to the University of Maryland IRB, and other parties annually. Amendments will also be provided to and approved by these parties.

12.3. Informed Consent Process

This process will include providing basic information in the local language about the purposes, eligibility, procedures, and risks of study participation. Parents will be encouraged to ask questions and answers will be provided as needed. This information will include the purpose of the trial, what will be asked of the participant and family, risks of the study, blood draw and storage of blood. The voluntary nature of the study will be emphasized and the right to withdraw for any reason without prejudice will be explained.

The consent form will be signed, or a thumbprint provided by the child’s parent or guardian before any procedures are conducted. A copy will be given to the participant, or his/her guardian and a copy will be stored at the study site.

If a parent or guardian is unable to read or sign the consent form, an impartial witness will assist for the entire informed consent process. The parent or guardian will provide a thumbprint, and the impartial witness will sign the consent form, thereby confirming that all information was provided,

and consent was freely given. The impartial witness can be a family or community member who is independent from the study team.

12.4. Subject Confidentiality

The study records will be stored in a locked office or in a password-protected secure database. No information concerning the study, or the data will be released to any unauthorized third party.

The study monitor or other authorized representatives of the sponsor may inspect all documents and records.

12.5. Study Discontinuation

Study discontinuation is not expected to occur. However, if the study is discontinued for safety reasons, parents of participants will be informed of the reasons for discontinuation and of the implications/potential consequences for the child. The study and its team will provide care for any medical needs of a participant as a result of the conduct of this study.

12.6. Sharing Study Results

When the clinical study report is completed, the investigators will share the summary results with the participating communities. Community meetings will be held to share the results of the study and to allow for questions and answers. It is anticipated that the results of this trial may have a significant bearing on policy decisions regarding the licensure of typhoid conjugate vaccines in Burkina Faso and other countries in Africa.

12.7. Use of Stored Specimens

Biological specimens will be maintained until the end of the study. This is to allow time for all study-related testing. The specimens will be maintained at the GRAS laboratory in Ouagadougou, Burkina Faso, and the University of Maryland CVD. No personal-identifying information associated with the study will be stored on site with the specimens. Following completion of the study, all specimens will be kept indefinitely in a secure location. If, before destruction, future unrelated studies are proposed for the specimens, such studies will be initiated with appropriate ethics committee approvals, and, if deemed appropriate by such committees, informed consent from participants.

12.8. Compensation

The parent/guardian of participants in this study will be given money for their time spent with the child during study visits. Participants will receive $15 (7500 FCFA) for each clinic visit.

13. Data Handling and Record Keeping

13.1. Source Data and Access to Data

Research records generated in this study will be stored in a locked room and on a secure electronic database. Only authorized personnel will have access to these data. Authorized

representatives of regulatory agencies may examine clinical records for the purposes of quality assurances reviews, audits, and evaluation of the study safety and progress.

13.2. Data Recording and Record Keeping

Data (safety and immune response data) will be recorded on paper CRFs for scanning into an electronic data base or entered directly into an electronic data based. CRFs designed specifically to capture data for this study will be generated. The study records will be stored in a locked office or in a password-protected secure database. The study records will be stored indefinitely by the principal investigator.

13.3. Procedures for Reporting Protocol Deviations

Protocol deviations will be collected in a CRF and kept in the study record. Deviations that affect the scientific integrity of the study or the rights/safety of a volunteer will be reported to University of Maryland and local IRBs according to the local regulations.

14. Publication Policy

The principal investigator and sponsor investigator will oversee collection, approvals, and dissemination of data from this study. All publications, including manuscripts, abstracts, oral/slide presentations, book chapters, etc. based on data collected in this study will be reviewed by each co-investigator before submission. Authors will acknowledge that the study was funded by the Bill & Melinda Gates Foundation (BMGF). In accordance with BMGF, all publications related to this study will be open access. Authorship will be determined in accordance with the International Committee of Medical Journal Editors guidelines and other contributors will be acknowledged.

15. Timeframe and Duration of the Study

15.1. Timeline

The total duration of the project is projected to take approximately 9 months, including approximately 2 months of patient recruitment and data collection.

15.2. Gantt Chart

	2024								
	Mar	Apr	May	Jun	July	Aug	Sep	Oct	Nov
**Activity**									
Protocol development	■								
IRB approval		■							
CRF and SOP development		■	■						
Participants recruitment & follow up				■	■	■	■		
Immunological assays						■	■	■	
Manuscript and results dissemination									■

References

1. Global Burden of Disease Collaborative Network. Global Burden of Disease, Typhoid Fever — Level 4 Cause. 2020. http://www.healthdata.org/results/gbd_summaries/2019/typhoid-fever-level-4-cause (accessed Aug 1 2021).
2. Patel PD, Liang Y, Meiring JE, et al. Efficacy of typhoid conjugate vaccine: final analysis of a 4-year, phase 3, randomised controlled trial in Malawian children. *Lancet* 2024.
3. Marks F, von Kalckreuth V, Aaby P, et al. Incidence of invasive *Salmonella* disease in sub-Saharan Africa: a multicentre population-based surveillance study. *Lancet Glob Health* 2017; **5**: e310-23.
4. Crump JA, Luby SP, Mintz ED. The global burden of typhoid fever. *Bulletin of the World Health Organization* 2004; **82**(5): 346-53.
5. Blum LS, Dentz H, Chingoli F, et al. Formative investigation of acceptability of typhoid vaccine during a typhoid fever outbreak in Neno District, Malawi. *Am J Trop Med Hyg* 2014; **91**(4): 729-37.
6. Kabwama SN, Bulage L, Nsubuga F, et al. A large and persistent outbreak of typhoid fever caused by consuming contaminated water and street-vended beverages: Kampala, Uganda, January - June 2015. *BMC Public Health* 2017; **17**(1): 23.
7. Polonsky JA, Martinez-Pino I, Nackers F, et al. Descriptive epidemiology of typhoid fever during an epidemic in Harare, Zimbabwe, 2012. *PLoS One* 2014; **9**(12): e114702.
8. Hendriksen RS, Leekitcharoenphon P, Lukjancenko O, et al. Genomic signature of multidrug-resistant Salmonella enterica serovar typhi isolates related to a massive outbreak in Zambia between 2010 and 2012. *J Clin Microbiol* 2015; **53**(1): 262-72.

9. Muyembe-Tamfum JJ, Veyi J, Kaswa M, Lunguya O, Verhaegen J, Boelaert M. An outbreak of peritonitis caused by multidrug-resistant Salmonella Typhi in Kinshasa, Democratic Republic of Congo. *Travel Med Infect Dis* 2009; **7**(1): 40-3.
10. World Health Organization. Typhoid vaccines: WHO position paper. *Wkly Epidemiol Rec* 2008; **83**(6): 49-59.
11. Thiem VD, Lin FY, Canh DG, et al. The Vi conjugate typhoid vaccine is safe, elicits protective levels of IgG anti-Vi, and is compatible with routine infant vaccines. *Clin Vaccine Immunol* 2011; **18**(5): 730-5.
12. Mohan VK, Varanasi V, Singh A, et al. Safety and immunogenicity of a Vi polysaccharide-tetanus toxoid conjugate vaccine (Typbar-TCV) in healthy infants, children, and adults in typhoid endemic areas: a multicenter, 2-cohort, open-label, double-blind, randomized controlled phase 3 study. *Clin Infect Dis* 2015; **61**(3): 393-402.
13. Gibani MM, Jin C, Darton TC, Pollard AJ. Control of Invasive Salmonella Disease in Africa: Is There a Role for Human Challenge Models? *Clin Infect Dis* 2015; **61 Suppl 4**: S266-71.
14. Jin C. Data from VAST trial - manuscript in progress.
15. Organization WH. Weekly Epidemiological Record. Geneva, Switzerland, 2017.
16. Immunizations GAfVa. Millions of children set to be protected against typhoid fever. Vientiane; 2017.
17. Organization WH. Typhoid Vaccine Prequalified. Geneva, Switzerland; 2018.
18. Sirima SB, Ouedraogo A, Barry N, et al. Safety and immunogenicity of co-administration of meningococcal type A and measles-rubella vaccines with typhoid conjugate vaccine in children aged 15-23 months in Burkina Faso. *Int J Infect Dis* 2021; **102**: 517-23.
19. van Damme P, Kafeja F, Anemona A, et al. Safety, immunogenicity and dose ranging of a new Vi-CRM_{197} conjugate vaccine against typhoid fever: randomized clinical testing in healthy adults. *PLoS One* 2011; **6**(9): e25398.
20. Biological E. Typhoid Vi Conjugate Vaccine Typhoid Conjugate Vaccine (Monovalent) TYPHIBEV. 2020.
21. Patel PD, Patel P, Liang Y, et al. Safety and Efficacy of a Typhoid Conjugate Vaccine in Malawian Children. *N Engl J Med* 2021; **385**(12): 1104-15.
22. Shakya M, Colin-Jones R, Theiss-Nyland K, et al. Phase 3 efficacy analysis of a typhoid conjugate vaccine trial in Nepal. *N Engl J Med* 2019; **381**(23): 2209-18.
23. Shakya M, Voysey M, Theiss-Nyland K, et al. Efficacy of typhoid conjugate vaccine in Nepal: final results of a phase 3, randomised, controlled trial. *Lancet Glob Health* 2021; **9**(11): e1561-e8.
24. Qadri F, Khanam F, Liu X, et al. Protection by vaccination of children against typhoid fever with a Vi-tetanus toxoid conjugate vaccine in urban Bangladesh: a cluster-randomised trial. *Lancet* 2021; **398**(10301): 675-84.
25. Sirima SB, Mordmuller B, Milligan P, et al. A phase 2b randomized, controlled trial of the efficacy of the GMZ2 malaria vaccine in African children. *Vaccine* 2016; **34**(38): 4536-42.
26. Sirima SB, Ouedraogo A, Barry N, et al. Safety and immunogenicity of Vi-typhoid conjugate vaccine co-administration with routine 9-month vaccination in Burkina Faso: A randomized controlled phase 2 trial. *Int J Infect Dis* 2021; **108**: 465-72.
27. Nampota-Nkomba N, Nyirenda OM, Khonde L, et al. Safety and immunogenicity of a typhoid conjugate vaccine among children aged 9 months to 12 years in Malawi: a nested substudy of a double-blind, randomised controlled trial. *Lancet Glob Health* 2022; **10**(9): e1326-e35.

28. Khanam F, Babu G, Rahman N, et al. Immune responses in children after vaccination with a typhoid Vi-tetanus toxoid conjugate vaccine in Bangladesh. *Vaccine* 2023; **41**(19): 3137-40.
29. Levine MM, Herrington D, Murphy JR, et al. Safety, infectivity, immunogenicity, and in vivo stability of two attenuated auxotrophic mutant strains of Salmonella typhi, 541Ty and 543Ty, as live oral vaccines in humans. *J Clin Invest* 1987; **79**(3): 888-902.
