# Supplementary material for: Immunogenicity and tolerability of a heterologous booster typhoid conjugate vaccine (TCV) five to six years after primary vaccination in Burkinabe Children: An open-label, non-randomized follow-up of a phase 2 trial cohort
Source: PLOS Glob Public Health. 2026 Nov 13;6(11):e0006855. doi: 10.1371/journal.pgph.0006855 (PMC13766224; doi:10.1371/journal.pgph.0006855)
Supplement: Supplementary file 2 [file pgph.0006855.s002.pdf]

S1_Table: Summary of unsolicited AEs by vaccine arm in the intention to treat population after Vi-CRM vaccination

	Booster TCV n=87	First TCV n=60	Overall n=147
**Abdominal pain**	1 (1.2)	1	2 (1.4)
**Angina**	0 (0.0)	1	1 (0.7)
**Cough**	1 (1.2)	0	1 (0.7)
**Diarrhea**	2 (2.3)	1	3 (2.0)
**Fever**	2 (2.3)	2	4 (2.7)
**Furunculosis**	0 (0.0)	1	1 (0.7)
**Headache**	4 (4.6)	2	6 (4.1)
**Rhinitis**	5 (5.8)	4 (6.7)	9 (6.1)
**Stomachache**	0 (0.0)	2 (3.3)	2 (1.4)
**Toothache**	0 (0.0)	1 (1.7)	1 (0.7)
**Uncomplicated Malaria**	7(8.0)	4 (6.7)	11 (7.5)
**Vomiting**	1 (1.2)	0 (0.0)	1 (0.7)
**Total**	23 (26.4)	19 (31.7)	42 (28.6)

Data are n (%). n=number of participants.
