# Supplementary material for: Immunogenicity and tolerability of a heterologous booster typhoid conjugate vaccine (TCV) five to six years after primary vaccination in Burkinabe Children: An open-label, non-randomized follow-up of a phase 2 trial cohort
Source: PLOS Glob Public Health. 2026 Nov 13;6(11):e0006855. doi: 10.1371/journal.pgph.0006855 (PMC13766224; doi:10.1371/journal.pgph.0006855)
Supplement: Supplementary file 3 [file pgph.0006855.s003.pdf]

S2_Table: Enrolled vs. Non-enrolled Children

	Enrolled (n=147)	Non-enrolled (n=103)	p-value
	n (%)	n (%)	
Original Trial Arm			1.0*
TCV	87 (59.2)	61 (59.2)	
IPV	60 (40.8)	42 (40.8)	
Age-Cohort			0.005*
9-11 months	48 (32.7)	52 (50.5)	
15-23 months	99 (67.3)	51 (49.5)	
Sex			0.81*
Female	72 (49.0)	52 (50.5)	
Male	75 (51.0)	51(49.5)	
Height mean(std)	73.8 (4.7)	72.7 (5.0)	0.07[#]
Weight mean (std)	9.1 (1.2)	8.9 (1.3)	0.21[#]
Temperature mean(std)	36.5 (0.36)	36.5 (0.31)	0.23[#]
Original immunogenicity sub-study participation			N/A
Yes	147 (100.0)	103 (100.0)	
No	0 (0.0)	0 (0.0)	
