# Supplementary material for: Immunogenicity and tolerability of a heterologous booster typhoid conjugate vaccine (TCV) five to six years after primary vaccination in Burkinabe Children: An open-label, non-randomized follow-up of a phase 2 trial cohort
Source: PLOS Glob Public Health. 2026 Nov 13;6(11):e0006855. doi: 10.1371/journal.pgph.0006855 (PMC13766224; doi:10.1371/journal.pgph.0006855)
Supplement: Supplementary file 4 [file pgph.0006855.s004.pdf]

S3_Table: Immunogenicity by Age at Primary Vaccination

Anti-Vi IgG before and 28 days after Vi-CRM vaccination given 5-6 years after receipt of first Vi-TT or IPV at 9-11months of age in the intention to treat population

	Booster-TCV		1st-TCV	
	n or n/N	*Mean or % (95% CI)*	*n or n/N*	*Mean or % (95% CI)*
GMT (EU/mL)				
Day 0	27	12·0 (8·0-18·2)	21	4·8 (3·5-6·4)
Day 28	27	3573·4 (2716·2-4701·1)	20	1844·9 (1220·0-2790·0)
GMFR				
Day 0 to 28	27	296·7 (176·8-497·9)	20	382·3 (241·4-605·6)
Seroconversion ≥ 4-fold increase from				
Day 0 to 28	27/27	100·0 (87·2-100·0)	20/20	100·0 (83·2-100·0)

n=number of participants. CI=confidence interval. Vi-CRM: Vi polysaccharide diphtheria toxin conjugated vaccine. Vi-TT: Vi polysaccharide tetanus toxoid conjugate vaccine. IPV: inactivated polio virus. GMT: geometric mean toters. EU: ELISA units. GMFR: geometric mean fold rise.

Anti-Vi IgG before and 28 days after Vi-CRM vaccination given 5-6 years after receipt of first Vi-TT or IPV at 15-23months of age in the intention to treat population

	Booster-TCV		1st-TCV	
	n or n/N	*Mean or % (95% CI)*	*n or n/N*	*Mean or % (95% CI)*
GMT (EU/mL)				
Day 0	60	27·8 21·8-35·5)	39	5·9 (4·5-7·7)
Day 28	59	6070·4 (4877·6-7554·9)	38	2223·3 (1811·1-2729·4)
GMFR				
Day 0 to 28	59	215·9 (159·5-292·0)	38	372·0 (297·4-465·3)
Seroconversion ≥ 4-fold increase from				
Day 0 to 28	59/59	100·0 (93·9-100·0)	38/38	100·0 (90·8-100·0)

n=number of participants. CI=confidence interval. Vi-CRM: Vi polysaccharide diphtheria toxin conjugated vaccine. Vi-TT: Vi polysaccharide tetanus toxoid conjugate vaccine. IPV: inactivated polio virus. GMT: geometric mean toters. EU: ELISA units. GMFR: geometric mean fold rise.
