# Supplementary material for: Immunogenicity and tolerability of a heterologous booster typhoid conjugate vaccine (TCV) five to six years after primary vaccination in Burkinabe Children: An open-label, non-randomized follow-up of a phase 2 trial cohort
Source: PLOS Glob Public Health. 2026 Nov 13;6(11):e0006855. doi: 10.1371/journal.pgph.0006855 (PMC13766224; doi:10.1371/journal.pgph.0006855)
Supplement: Supplementary file 5 [file pgph.0006855.s005.docx]

**S1_CONSORT_Checklist. CONSORT 2025 Checklist**

**Checklist of information to include when reporting clinical trials**

*This CONSORT 2025 checklist is completed for a* ***non‑randomized, open‑label, prospective follow‑up interventional booster study*** *enrolling children previously vaccinated in a registered clinical trial (ClinicalTrials.gov identifier* ***NCT03614533****). Items not applicable due to study design are explicitly indicated.*

**Title and Abstract**

| **Section/Topic** | **Item No.** | **Checklist item** | **Location where item is reported** |
| --- | --- | --- | --- |
| Title | 1a | Identification of the study as a clinical trial | Title page |
| Abstract | 1b | Structured summary of background, objectives, methods, results, and conclusions | Abstract |
| Abstract | 1c | Trial registration number and registry name | Abstract (Trial Registration: ClinicalTrials.gov, NCT03614533) |

**Introduction**

| **Section/Topic** | **Item No.** | **Checklist item** | **Location where item is reported** |
| --- | --- | --- | --- |
| Background and rationale | 2a | Scientific background and explanation of rationale | Introduction |
| Objectives | 2b | Specific objectives or hypotheses | Introduction (final paragraph) |

**Methods**

**Trial design**

| **Section/Topic** | **Item No.** | **Checklist item** | **Location where item is reported** |
| --- | --- | --- | --- |
| Trial design | 3a | Description of trial design (e.g., follow‑up, non‑randomized, interventional) | Methods – Study design |
| Changes after commencement | 3b | Important changes to methods after trial commencement | Not applicable (no changes) |

**Participants**

| **Section/Topic** | **Item No.** | **Checklist item** | **Location where item is reported** |
| --- | --- | --- | --- |
| Eligibility criteria | 4a | Eligibility criteria for participants | Methods – Participants |
| Settings and locations | 4b | Settings and locations where data were collected | Methods – Study design |

**Interventions**

| **Section/Topic** | **Item No.** | **Checklist item** | **Location where item is reported** |
| --- | --- | --- | --- |
| Interventions | 5 | Sufficient details of the intervention to allow replication (vaccine, dose, route, timing) | Methods – Procedures |

**Outcomes**

| **Section/Topic** | **Item No.** | **Checklist item** | **Location where item is reported** |
| --- | --- | --- | --- |
| Outcomes | 6a | Prespecified primary and secondary outcome measures | Methods – Outcomes |
| Changes to outcomes | 6b | Any changes to outcomes after trial commencement | Not applicable (no changes) |

**Sample size**

| **Section/Topic** | **Item No.** | **Checklist item** | **Location where item is reported** |
| --- | --- | --- | --- |
| Sample size | 7a | How sample size was determined | Methods – Statistical analysis |
| Interim analyses | 7b | Interim analyses and stopping guidelines | Not applicable |

**Randomization**

**Items 8a–10 not applicable.**

**Explanation:** Participants were not randomized; this was a **non‑randomized follow‑up interventional booster study** enrolling participants based on vaccination history in a prior trial.

**Blinding (Masking)**

| **Section/Topic** | **Item No.** | **Checklist item** | **Location where item is reported** |
| --- | --- | --- | --- |
| Blinding | 11a | Who was blinded after assignment to intervention | Not applicable (open‑label study) |
| Blinding | 11b | Description of similarity of interventions | Not applicable |

**Statistical methods**

| **Section/Topic** | **Item No.** | **Checklist item** | **Location where item is reported** |
| --- | --- | --- | --- |
| Statistical methods | 12a | Statistical methods used for primary and secondary outcomes | Methods – Statistical analysis |
| Additional analyses | 12b | Methods for additional analyses (e.g., multi‑time‑point analysis) | Methods – Statistical analysis |

**Results**

**Participant flow**

| **Section/Topic** | **Item No.** | **Checklist item** | **Location where item is reported** |
| --- | --- | --- | --- |
| Participant flow | 13a | Numbers of participants at each stage of the study | Figure 1 |
| Losses and exclusions | 13b | Losses and exclusions after enrollment, with reasons | Figure 1 |

**Recruitment**

| **Section/Topic** | **Item No.** | **Checklist item** | **Location where item is reported** |
| --- | --- | --- | --- |
| Recruitment | 14a | Dates defining enrollment and follow‑up periods | Results – Baseline characteristics |
| Trial termination | 14b | Reason for trial termination | Not applicable (completed as planned) |

**Baseline data**

| **Section/Topic** | **Item No.** | **Checklist item** | **Location where item is reported** |
| --- | --- | --- | --- |
| Baseline characteristics | 15 | Baseline demographic and clinical characteristics of participants | Table 1 |

**Numbers analyzed**

| **Section/Topic** | **Item No.** | **Checklist item** | **Location where item is reported** |
| --- | --- | --- | --- |
| Analysis populations | 16 | Number of participants included in each analysis | Results; Table 2; Table 3 |

**Outcomes and estimation**

| **Section/Topic** | **Item No.** | **Checklist item** | **Location where item is reported** |
| --- | --- | --- | --- |
| Outcomes | 17a | Results for each primary and secondary outcome | Results – Immunogenicity |
| Precision | 17b | Precision of estimates (e.g., 95% confidence intervals) | Tables 2 and 4; Figures 2 and 3 |

**Ancillary analyses**

| **Section/Topic** | **Item No.** | **Checklist item** | **Location where item is reported** |
| --- | --- | --- | --- |
| Additional analyses | 18 | Results of additional analyses, including longitudinal analyses | Results – Multi‑time‑point analysis  Figure 3 |

**Harms**

| **Section/Topic** | **Item No.** | **Checklist item** | **Location where item is reported** |
| --- | --- | --- | --- |
| Adverse events | 19 | All important harms or unintended effects in each group | Results – Safety; Table 2 |

**Discussion**

| **Section/Topic** | **Item No.** | **Checklist item** | **Location where item is reported** |
| --- | --- | --- | --- |
| Limitations | 20 | Trial limitations and potential sources of bias | Discussion |
| Generalizability | 21 | Generalizability (external validity) of findings | Discussion |
| Interpretation | 22 | Interpretation consistent with results and existing evidence | Discussion |

**Other Information**

| **Section/Topic** | **Item No.** | **Checklist item** | **Location where item is reported** |
| --- | --- | --- | --- |
| Trial registration | 23 | Trial registration number and name of registry | Abstract; Methods |
| Protocol access | 24 | Where the full trial protocol can be accessed | Supporting Information |
| Funding | 25 | Sources of funding and role of the funders | Funding statement, financial disclosure |

**Footnote**

- Trial registration: **ClinicalTrials.gov, NCT03614533**
- Study type: Post‑licensure, non‑randomized, open‑label, prospective follow‑up interventional booster study
- Reporting guideline: **CONSORT 2025 (adapted)**
