# Supplementary material for: Immunogenicity and tolerability of a heterologous booster typhoid conjugate vaccine (TCV) five to six years after primary vaccination in Burkinabe Children: An open-label, non-randomized follow-up of a phase 2 trial cohort
Source: PLOS Glob Public Health. 2026 Nov 13;6(11):e0006855. doi: 10.1371/journal.pgph.0006855 (PMC13766224; doi:10.1371/journal.pgph.0006855)
Supplement: Supplementary file 6 [file pgph.0006855.s006.docx]

**SPIRIT 2025 Checklist**

**Standard Protocol Items: Recommendations for Interventional Trials (SPIRIT 2025)**

*Completed SPIRIT 2025 checklist for a non‑randomized, open‑label, prospective follow‑up interventional booster study enrolling participants from a previously registered clinical trial (ClinicalTrials.gov identifier NCT03614533). Items not applicable due to study design are explicitly noted.*

**Administrative Information**

| **Section/Topic** | **Item No.** | **Description** | **Location where addressed** |
| --- | --- | --- | --- |
| Title | 1 | Descriptive title identifying the study design and population | Protocol title page |
| Trial registration | 2a | Trial identifier and registry name | Protocol; ClinicalTrials.gov NCT03614533 |
| WHO data set | 2b | Items from WHO Trial Registration Data Set | ClinicalTrials.gov record |
| Protocol version | 3 | Date and version identifier of protocol | Protocol version/date |
| Funding | 4 | Sources and types of financial support | Protocol; Funding statement |
| Roles and responsibilities | 5a | Names and roles of protocol contributors | Protocol – Administrative section |
|  | 5b | Name of sponsor and funders | Protocol; Funding statement |
|  | 5c | Role of sponsor/funder in study design and conduct | Protocol; Funding statement |
|  | 5d | Composition and role of oversight committees | Not applicable (no DSMB required) |

**Introduction**

| **Section/Topic** | **Item No.** | **Description** | **Location** |
| --- | --- | --- | --- |
| Background and rationale | 6a | Description of research question and justification | Protocol – Introduction |
| Comparators | 6b | Explanation for choice of comparators | Protocol – Study design |
| Objectives | 7 | Specific objectives and hypotheses | Protocol – Objectives |
| Trial design | 8 | Description of trial design (non‑randomized, follow‑up, open‑label) | Protocol – Study design |

**Methods: Participants, Interventions, and Outcomes**

| **Section/Topic** | **Item No.** | **Description** | **Location** |
| --- | --- | --- | --- |
| Study setting | 9 | Description of study sites and settings | Protocol – Study design |
| Eligibility criteria | 10 | Inclusion and exclusion criteria | Protocol – Participants |
| Interventions | 11a | Detailed description of intervention (vaccine, dose, schedule) | Protocol – Procedures |
|  | 11b | Criteria for discontinuing or modifying intervention | Protocol – Safety |
|  | 11c | Strategies to improve adherence | Not applicable (single‑dose intervention) |
|  | 11d | Concomitant care and interventions | Protocol – Procedures |
| Outcomes | 12 | Definition of primary and secondary outcomes | Protocol – Outcomes |
| Participant timeline | 13 | Schedule of enrollment, intervention, and assessments | Protocol – Schedule of assessments |
| Sample size | 14 | Rationale for sample size | Protocol – Statistical considerations |
| Recruitment | 15 | Strategies for participant recruitment | Protocol – Recruitment |

**Methods: Assignment of Interventions**

| **Section/Topic** | **Item No.** | **Description** | **Location** |
| --- | --- | --- | --- |
| Allocation | 16a | Method of allocation sequence generation | Not applicable (non‑randomized) |
| Concealment | 16b | Allocation concealment mechanism | Not applicable |
| Implementation | 16c | Implementation of allocation sequence | Not applicable |
| Blinding | 17a | Who is blinded | Not applicable (open‑label) |
|  | 17b | Circumstances for unblinding | Not applicable |

**Methods: Data Collection, Management, and Analysis**

| **Section/Topic** | **Item No.** | **Description** | **Location** |
| --- | --- | --- | --- |
| Data collection | 18a | Plans for outcome data collection | Protocol – Data collection |
|  | 18b | Data quality control (training, monitoring) | Protocol – Study procedures |
| Data management | 19 | Data entry, coding, security, and storage | Protocol – Data management |
| Statistical methods | 20a | Statistical methods for primary and secondary outcomes | Protocol – Statistical analysis |
|  | 20b | Methods for additional analyses | Protocol – Statistical analysis |
|  | 20c | Definition of analysis populations and missing data handling | Protocol – Statistical analysis |

**Methods: Monitoring**

| **Section/Topic** | **Item No.** | **Description** | **Location** |
| --- | --- | --- | --- |
| Data monitoring | 21a | Data Monitoring Committee composition and role | Not applicable |
|  | 21b | Interim analyses and stopping guidelines | Not applicable |
| Harms | 22 | Plans for adverse event collection and reporting | Protocol – Safety |
| Auditing | 23 | Plans for trial auditing | Not applicable |

**Ethics and Dissemination**

| **Section/Topic** | **Item No.** | **Description** | **Location** |
| --- | --- | --- | --- |
| Ethics approval | 24 | Research ethics committee approval | Protocol – Ethics |
| Protocol amendments | 25 | Plans for communicating protocol amendments | Protocol – Amendments |
| Consent | 26a | Consent process | Protocol – Informed consent |
|  | 26b | Ancillary consent provisions | Not applicable |
| Confidentiality | 27 | Protection of participant confidentiality | Protocol – Data protection |
| Conflicts of interest | 28 | Declaration of competing interests | Protocol; Manuscript |
| Access to data | 29 | Data access arrangements | Protocol – Data sharing |
| Ancillary care | 30 | Provisions for ancillary care | Protocol – Safety |
| Dissemination policy | 31a | Plans for communicating trial results | Protocol – Dissemination |
|  | 31b | Authorship guidelines | Protocol – Dissemination |
|  | 31c | Public access to protocol and data | Protocol – Data sharing |

**Appendices**

| **Section** | **Item No.** | **Description** | **Location** |
| --- | --- | --- | --- |
| Informed consent | 32 | Model consent form | Protocol appendix |
| Biological specimens | 33 | Plans for specimen collection and use | Protocol – Laboratory methods |

**Footnote**

- Trial registration: **ClinicalTrials.gov, NCT03614533**
- Study type: Post‑licensure, non‑randomized, open‑label, prospective follow‑up interventional booster study
- Reporting guideline: **SPIRIT 2025 (adapted)**
